# Microglia ferroptosis is regulated by SEC24B and contributes to neurodegeneration

**DOI:** 10.1038/s41593-022-01221-3

**Published:** 2022-12-19

**Authors:** Sean K. Ryan, Matija Zelic, Yingnan Han, Erin Teeple, Luoman Chen, Mahdiar Sadeghi, Srinivas Shankara, Lilu Guo, Cong Li, Fabrizio Pontarelli, Elizabeth H. Jensen, Ashley L. Comer, Dinesh Kumar, Mindy Zhang, Joseph Gans, Bailin Zhang, Jonathan D. Proto, Jacqueline Saleh, James C. Dodge, Virginia Savova, Deepak Rajpal, Dimitry Ofengeim, Timothy R. Hammond

**Affiliations:** 1 Sanofi, Rare and Neurologic Diseases, Cambridge, MA USA; 2 Sanofi, Precision Medicine and Computational Biology, Cambridge, MA USA

**Keywords:** Neurodegeneration, Neuroimmunology, Microglia, Parkinson's disease, Cell death

## Abstract

Iron dysregulation has been implicated in multiple neurodegenerative diseases, including Parkinson’s disease (PD). Iron-loaded microglia are frequently found in affected brain regions, but how iron accumulation influences microglia physiology and contributes to neurodegeneration is poorly understood. Here we show that human induced pluripotent stem cell-derived microglia grown in a tri-culture system are highly responsive to iron and susceptible to ferroptosis, an iron-dependent form of cell death. Furthermore, iron overload causes a marked shift in the microglial transcriptional state that overlaps with a transcriptomic signature found in PD postmortem brain microglia. Our data also show that this microglial response contributes to neurodegeneration, as removal of microglia from the tri-culture system substantially delayed iron-induced neurotoxicity. To elucidate the mechanisms regulating iron response in microglia, we performed a genome-wide CRISPR screen and identified novel regulators of ferroptosis, including the vesicle trafficking gene *SEC24B*. These data suggest a critical role for microglia iron overload and ferroptosis in neurodegeneration.

## Abstract (web summary)

Iron-laden microglia assume a disease-relevant, ferroptosis-associated signature and cause neurotoxicity. CRISPR screen uncovered regulators of ferroptosis in microglia. This ferroptosis–microglia–neurodegeneration axis could be targeted therapeutically.

## Main

Iron is the most abundant transition metal in the brain^1^. Disrupted iron homeostasis^2^ is correlated with disease progression in several neurodegenerative disorders, including Parkinson’s disease (PD) and amyotrophic lateral sclerosis (ALS)^3,4^. Microglia have one of the highest iron storage capacities of all brain cell types and accumulate iron in disease^1,5–9^, but how iron overload impacts microglia function and whether iron-laden microglia contribute to neurodegeneration is not well understood.

Microglia could also be susceptible to an iron-dependent form of cell death called ferroptosis^10,11^. Ferroptosis is distinct from other forms of cell death, such as apoptosis and necroptosis, and is driven by iron-dependent phospholipid peroxidation^10^. Ferroptosis has been implicated in multiple neurodegenerative disorders, including PD, and mutations in the iron storage gene *FTL* cause a rare form of Parkinsonism^12^. Disease-relevant subtypes of neurons are susceptible to ferroptosis^13,14^, but the role in glia is largely unexplored.

To understand the role of iron accumulation in human microglia and to model the complex interactions between neurons and glia in a disease-relevant system, we used a human induced pluripotent stem cell (iPSC)-derived tri-culture system that contains microglia, neurons and astrocytes^15^. We found that microglia had the strongest transcriptional response to iron dysregulation among the three cell types, and we identify a subset of microglia with a distinct ferroptosis-associated transcriptomic signature (FAS) that is enriched in the postmortem ALS spinal cord and the postmortem PD patient midbrain microglia. In the tri-culture system, removal of microglia reduced neuronal lipid peroxidation and significantly delayed neuronal death under ferroptotic conditions. To identify ferroptosis regulators in microglia, we performed a genome-wide CRISPR screen and uncovered a novel ferroptosis susceptibility gene, *SEC24B*. Finally, we performed a small-molecule screen to identify inhibitors of microglia ferroptosis and showed that pharmacological modulation may be a viable strategy to mitigate ferroptosis in neurodegenerative disease. Together, these findings identify a cell non-autonomous role for microglia in ferroptosis-driven neurodegeneration.

## Results

### Human iPSC-derived tri-culture is susceptible to ferroptosis

**Fig. 1:**
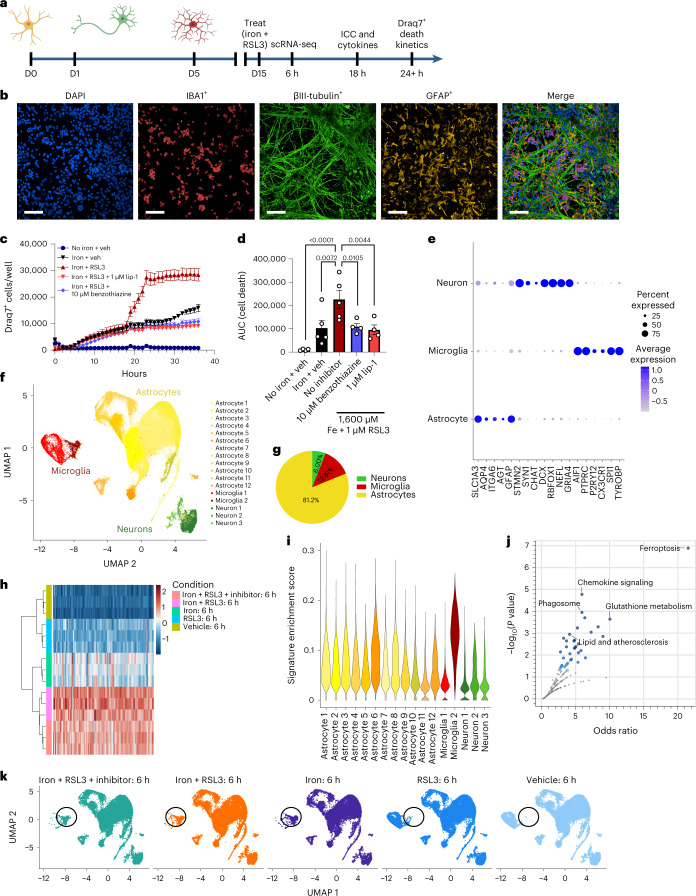
Ferroptosis induction causes a unique transcriptional response and cell death in iPSC tri-cultures. **a**, Schema for generation of tri-culture and downstream analysis. Illustrations were created with BioRender. **b**, Representative image of tri-culture showing IBA1^+^ (red) microglia, βIII-tubulin^+^ (green) neurons and GFAP^+^ (yellow) astrocytes. Scale bar, 100 µm (*n* = 3 biologically independent experiments). **c**, Draq7^+^ death kinetics in tri-cultures exposed to 1,600 µM iron + 1 µM RSL3 ± 1 µM lip-1 or 10 µM benzothiazine. Representative graph (*n* = 1 biologically independent experiment). Error bars represent s.e.m. of the technical replicates. **d**, Area under the curve (AUC) of death kinetics 12 hours after separation from iron + veh condition (*n* = 4 biologically independent experiments). Two-way ANOVA, Dunnett post hoc. *P* values are indicated in the graph. Error bars represent s.e.m. **e**, Dot plot of cell-type-specific genes for microglia, neurons and astrocytes. **f**, UMAP representation of scRNA-seq analysis of 109,100 cells from tri-cultures exposed to vehicle or 1,600 µM iron + 1 µM RSL3 ± commercial ferroptosis inhibitors. **g**, Pie chart for percentages of each cell type in scRNA-seq collection. **h**, Pseudo-bulk analysis of all cell types and heat map of top dysregulated genes. Inhibitor was 10 µM benzothiazine (*n* = 3 biologically independent experiments). **i**, Violin plot of iron + RSL3-induced signature enrichment UCell score for all 17 clusters using the top 50 genes from **h**. **j**, KEGG pathway analysis of top 200 DEGs in iron + RSL3-induced signature. Fisher exact test. **k**, UMAP colored by treatment condition. D, day; veh, vehicle.

**Extended Data Fig. 1:**
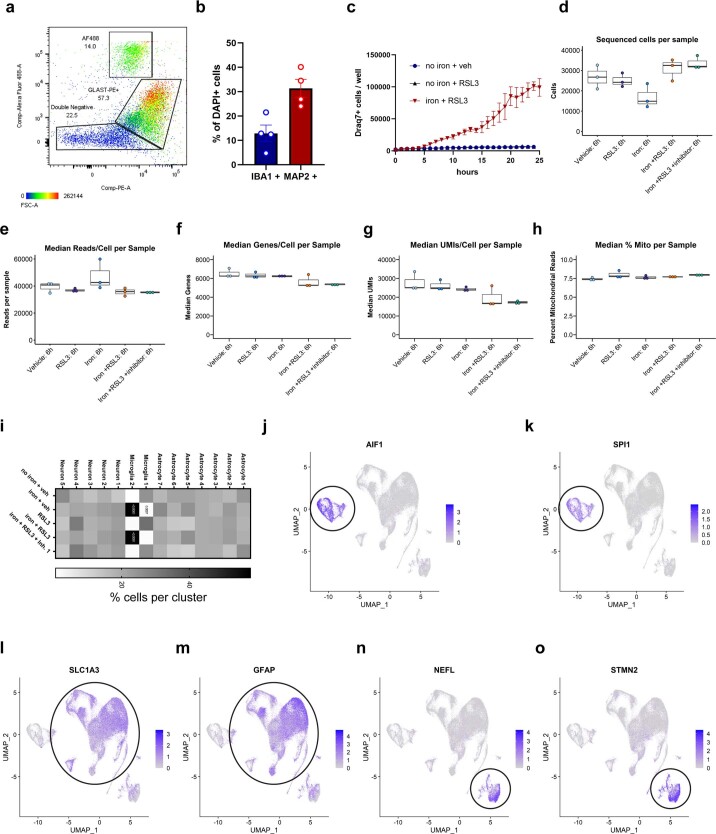
Tri-culture cell type validation and death kinetics of RSL3 only. **a**, Flow cytometry analysis isolating GFP+ microglia, GLAST-PE+ astrocytes, and double-negative neurons. (n = 1). **b**, Quantification of percent IBA1+ and MAP2+ cells by ICC in tri-culture (n = 4 biologically independent experiments). Error bars represent SEM. **c**, Death kinetics of no iron + RSL3 compared to no iron + veh and iron + RSL3. (n = 3 biologically independent experiments). Error bars represent SEM. **d–h**, quality control for scRNAseq. Minima = minimum value, maxima = maximum value, center = median, bounds of box = 25^th^ and 75^th^ percentile, bounds of whisker = minima and maxima. **i**, Percent cells per cluster per condition. (n = 3 biologically independent experiments). Two-way ANOVA, Tukey post hoc. p-values are indicated in graph. **j–o**, Cell type identification of gene expression by **j, k** *AIF1* and *SPI1* for microglia, **l,m** *SLC1A3* and *GFAP* for astrocytes, and **n,o** *NEFL* and *STMN2* for neurons.

To better understand the role of iron signaling and ferroptosis in the brain, we established a human iPSC-derived tri-culture of neurons, astrocytes and microglia (Fig. 1a)^15,16^ that consisted of approximately 15% microglia, 25% neurons and 60% astrocytes as determined by fluorescence-activated cell sorting (FACS) and immunocytochemistry (Extended Data Fig. 1a,b). All cell types formed a complex network within 2 weeks (Fig. 1b). To study the role of iron overload and ferroptosis, we treated the cultures with iron and RSL3 (iron + RSL3), an inhibitor of GPX4 and known ferroptosis inducer^10^. Although iron or RSL3 alone led to minimal cell death as assessed by cell death dye Draq7, inhibition of GPX4 and exogenous iron induced robust cell death 20 hours after treatment, suggesting that this culture system can undergo ferroptosis (Fig. 1c,d and Extended Data Fig. 1c). In support of ferroptosis as the mechanism of death, iron + RSL3-induced cell death was inhibited to iron-alone levels by two antioxidant ferroptosis inhibitors: liproxstatin-1 (lip-1) and a pyrimido-benzothiazine derivative (benzothiazine)^17,18^ (Fig. 1c,d) and to vehicle levels by the iron chelator deferoxamine (DFO) (Fig. 3c). By 18 hours after treatment, there was significant microglia death, but neuronal death mostly occurred around 20 hours (Fig. 1c and Extended Data Fig. 3a,b,f). Ferroptosis in astrocytes was not observed at any timepoint by bright-field, possibly due to higher expression of the anti-ferroptotic NF2-Hippo pathway-related genes^19^ (Extended Data Fig. 3f,i), but further investigation of astrocyte resistance to ferroptosis is needed. These results show that the human tri-culture system can be used as a model of iron response and ferroptosis in cell types relevant to neurodegenerative disease.

To better elucidate how the different cell types responded to ferroptosis induction, we measured cell-specific transcriptomic changes in the tri-culture system by single-cell RNA sequencing (scRNA-seq). We analyzed the cells 6 hours after stimulation, a timepoint that precedes significant death induction in the iron + RSL3 condition (Fig. 1c). Transcription inhibitors were used to preserve cell state during the preparation of the cells^20^. In total, 109,100 cells were sequenced with a median depth of ~40,000 reads per cell (Extended Data Fig. 1e). We identified 17 clusters across the five conditions: vehicle-treated (no iron + veh), RSL3 only, iron only, iron + RSL3 and iron + RSL3 + benzothiazine (Fig. 1f and Extended Data Fig. 1d–i). Using cluster gene expression signatures and several known markers for each cell type, we were able to distinguish microglia, neurons and astrocytes across all conditions (Fig. 1e–g and Extended Data Fig. 1i–o). We performed pseudo-bulk analysis and identified a ferroptosis-induced signature in the iron + RSL3 condition compared to vehicle (Fig. 1h). Enrichment for the ferroptosis-induced signature was then examined across all 17 subclusters using UCell^21^. We found that microglia subcluster 2 had the largest signature enrichment per cell (Fig. 1i). KEGG pathway analysis^22^ of the signature identified ferroptosis and glutathione metabolism as the top affected pathways. In addition, several immune-related and myeloid-related pathways were dysregulated, including chemokine signaling and phagosome (Fig. 1j). These results show that microglia are most sensitive to iron overload compared to neurons and astrocytes and may be a key player in the ferroptosis cascade.

### Iron overload and ferroptosis alter microglia state

**Fig. 2:**
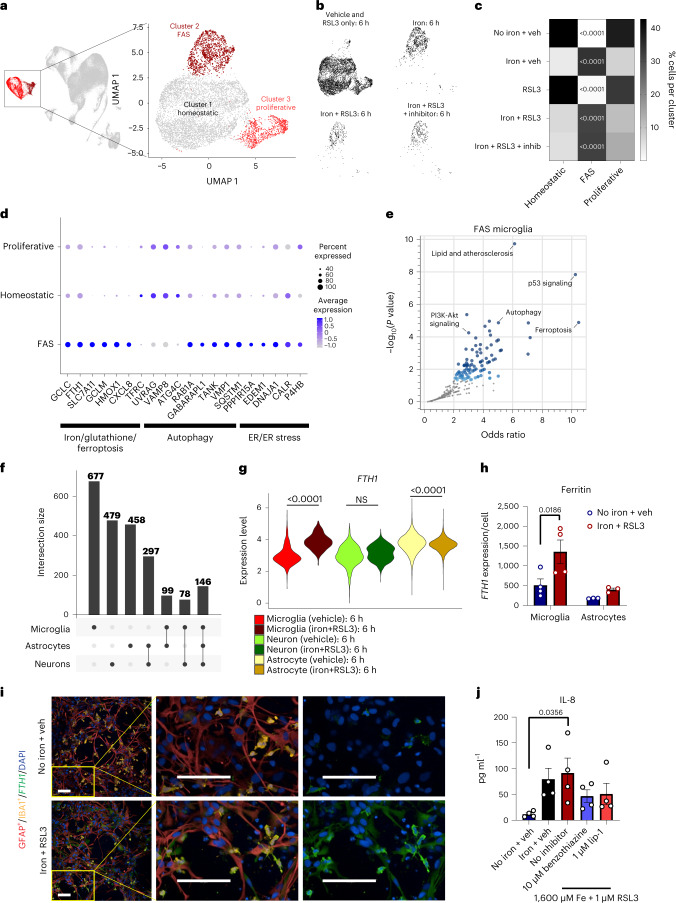
Ferroptosis induction causes a profound shift in microglia cell state compared to astrocytes and neurons. **a**,**b**, UMAP microglia subclusters (homeostatic, FAS and proliferative) (**a**) and plots for each treatment condition in black (**b**). **c**, Heat map of the normalized percent of cells from each sample assigned to each cluster. Two-way ANOVA, Tukey post hoc. *P* values are indicated in the graph. **d**, Select dysregulated genes associated with ferroptosis, autophagy or ER stress. **e**, KEGG pathway analysis of the top 200 upregulated and downregulated genes in FAS microglia cluster. Fisher exact test. **f**, Intersections of the top 1,000 dysregulated genes among microglia, neurons and astrocytes in iron + RSL3 versus vehicle. **g**, Violin plots for *FTH1* expression in microglia (red), neurons (green) and astrocytes (yellow) under vehicle or iron + RSL3 conditions. Wald test with DESeq2. NS, not significant. *P* values are indicated in the graph. **h**, Average expression of *FTH1* per IBA1^+^ microglia (*n* = 4 biologically independent experiments) or GFAP^+^ astrocyte (*n* = 3 biologically independent experiments). Log-transformed, two-way ANOVA, Sidak post hoc. *P* values are indicated in the graph. Error bars represent s.e.m. **i**, Representative images of *FTH1* (green) expression in IBA1^+^ microglia (yellow) and GFAP^+^ astrocytes (red) in tri-cultures 18 hours after treatment. Scale bar, 100 µm (*n* = 3 biologically independent experiments). **j**, IL-8 production among conditions (*n* = 4 biologically independent experiments). One-Way ANOVA, Dunnett post hoc. *P* values are indicated in the graph. Error bars represent s.e.m. NS, not significant; veh, vehicle.

**Extended Data Fig. 2:**
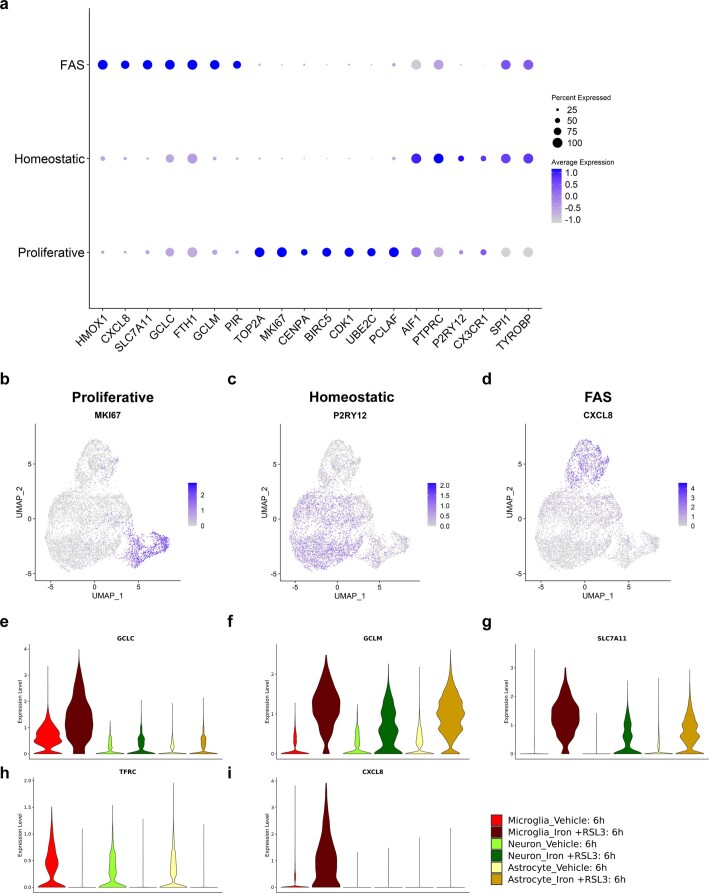
Characterization of microglia subclusters and ferroptosis-related genes across cell types. **a**, Dot plot of subcluster specific genes for FAS, homeostatic, and proliferative clusters. **b**, UMAP for expression pattern of proliferative marker *MKI67*. **c**, UMAP for expression pattern of homeostatic marker P2RY12. **d**, UMAP for expression pattern of FAS marker *CXCL8*. **e-i**, violin plots for ferroptosis-related genes (*GCLC, GCLM, SLC7A11, TFRC*, and *CXCL8*) in microglia, neurons, and astrocytes under vehicle or iron + RSL3 conditions.

To understand the effect of the ferroptotic stimuli directly on microglia cell state, we subclustered the microglia and uncovered three unique subpopulations (Fig. 2a–c and Extended Data Fig. 2a–d). By examining enriched genes and using KEGG pathway analysis, we identified cluster 2 as a unique FAS subpopulation. The cells in this cluster had enrichment for ferroptosis and lipid pathways as well as p53 signaling, autophagy and endoplasmic reticulum (ER) stress (Fig. 2d,e). The FAS subpopulation was highly enriched in the iron-only, iron + RSL3 and iron + RSL3 + benzothiazine conditions and had the highest enrichment score for the pseudo-bulk signature (Figs. 1i,k and 2a–c). FAS microglia persisted in the iron + RSL3 + benzothiazine condition, suggesting that this antioxidant inhibitor acts downstream of iron, despite largely preventing ferroptosis.

The other two microglia subpopulations, which were enriched in the control conditions, included a homeostatic subpopulation (cluster 1) and a proliferative subpopulation (cluster 3) (Fig. 2a,b and Extended Data Fig. 2a–c). These data demonstrate that microglia undergo a marked shift in cell state after exposure to iron and before cell death.

**Extended Data Fig. 3:**
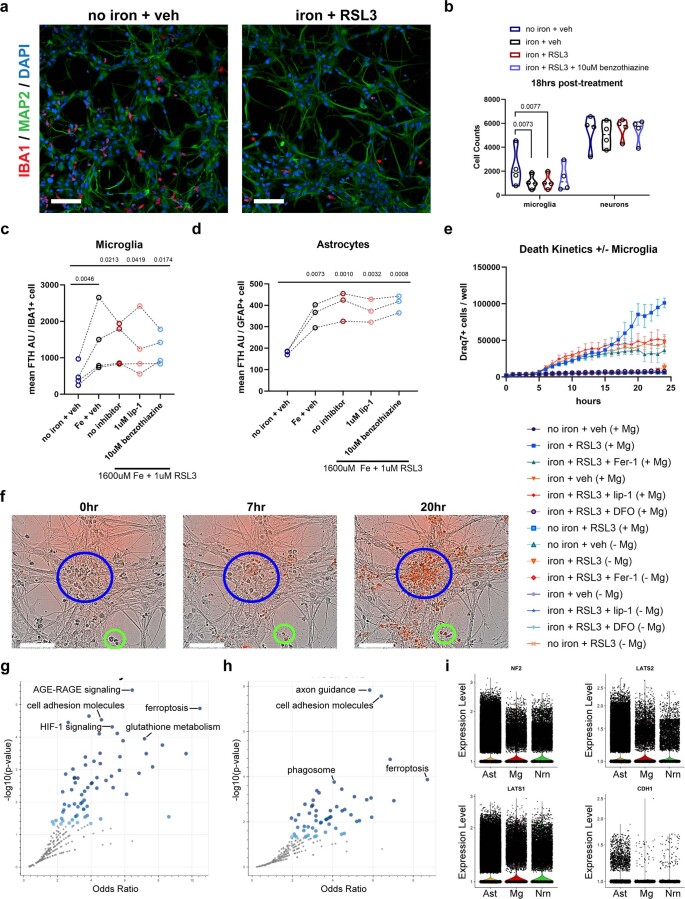
Ferroptosis is induced in microglia, followed by neurons. **a**, Representative images of IBA1+ microglia (red) and MAP2+ neurons (green) in tri-culture after 18 hr exposure to no iron + veh or iron + RSL3 treatments. Scale bar = 100 µm. **b**, Quantification of microglia and neurons 18hrs post-treatment (n = 4 biologically independent experiments). One-way ANOVA, Dunnett post hoc, log transformed. p-values are indicated in graph. **c**, Quantification of ferritin expression in microglia (n = 4 biologically independent experiments). Log transformed, RM one-way ANOVA, Dunnett post hoc. p-values are indicated in graph. **d**, Quantification of ferritin expression in astrocytes (n = 3 biologically independent experiments). One-way ANOVA, Dunnett post hoc. p-values are indicated in graph. **e**, Full death kinetics for all conditions of tri-cultures with and without microglia (n = 3 biologically independent experiments). Error bars represent SEM. **f**, Representative images of tri-culture treated with iron + RSL3 at 0 hr, 7hrs, and 20hrs post-treatment (n = 4 biologically independent experiments). Blue circle identifies a neuron cluster. Green circle identifies microglia by morphology. Red cells are Draq7+ cells, indicating microglia death by 7hrs and neuronal death around 20hrs post-treatment. Scale bar = 100 µm. **g**, KEGG pathway analysis of top 200 upregulated and downregulated genes in astrocytes in iron + RSL3 versus veh. Fisher exact test. **h**, KEGG pathway analysis of top 200 upregulated and downregulated genes in neurons in iron + RSL3 versus veh. Fisher exact test. **i**, violin plots for *NF2*, *LATS1*, *LATS2*, and *CHD1* in astrocytes (Ast), microglia (Mg), and neurons (Nrn).

Despite a clear microglia response to iron overload, we also examined the transcriptional response in neurons and astrocytes by calculating the differentially expressed genes (DEGs) between the iron + RSL3 and no iron + vehicle conditions in each cell type. Several of the same ferroptosis-related and iron-related genes that were upregulated in the FAS microglia were also differentially expressed in astrocytes and neurons, including *GCLC*, *GCLM*, *SLC7A11* and *TFRC*. However, *CXCL8*, a top marker for FAS microglia, was not expressed in the other cell types (Extended Data Fig. 2e–i), and overall, there was limited overlap of DEGs across the cell types (Fig. 2f). However, ferroptosis was a top activated pathway in neurons and astrocytes (Extended Data Fig. 3g,h). One of the top DEGs in the FAS microglia was the gene encoding the ferritin heavy chain, *FTH1* (Fig. 2d). Ferritin is the main protein that sequesters iron in the cell, and increased expression has been associated with ferroptosis^23^. *FTH1* was upregulated in microglia but unchanged in neurons and downregulated in astrocytes (Fig. 2g), suggesting differences in iron sequestration after exposure to ferroptosis-inducing stimuli. To confirm this, we performed immunocytochemistry and found that FTH protein levels were significantly increased in microglia, but not astrocytes, after exposure to iron + RSL3 (Fig. 2h,i and Extended Data Fig. 3c,d). These findings show that microglia have the greatest response to iron exposure and sequester the greatest amount per cell in the tri-culture^24^.

**Fig. 3:**
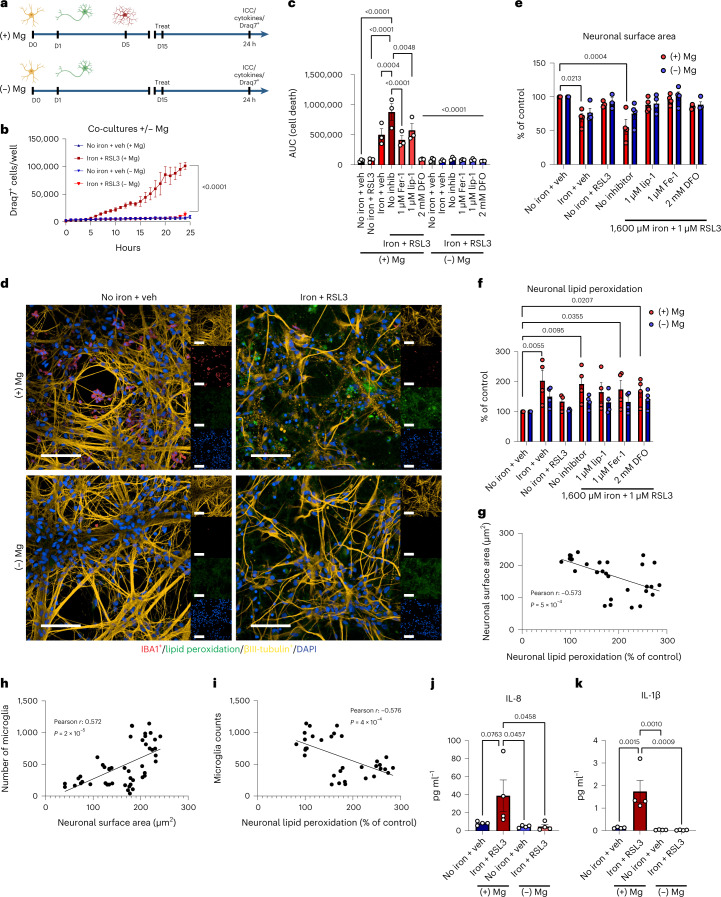
Microglia sensitize neurons to ferroptosis-dependent degeneration. **a**, Schema for co-cultures ± microglia. Illustrations were created with BioRender. **b**, Draq7^+^ death kinetics in tri-cultures exposed to 1,600 µM iron + 1 µM RSL3 ± microglia (*n* = 3 biologically independent experiments). Two-way ANOVA, Dunnett post hoc. *P* values are indicated in the graph. **c**, AUC of death kinetics (*n* = 3 biologically independent experiments). Two-way ANOVA, Dunnett post hoc. *P* values are indicated in the graph. Error bars represent s.e.m. **d**, Representative images of cultures ± microglia under vehicle or ferroptotic conditions. βIII-tubulin^+^ (yellow) neurons are dying and have increased lipid peroxidation (green) in cultures with IBA1^+^ (red) microglia (*n* = 4 biologically independent experiments). Scale bar, 100 µm. **e**, Average neuronal surface area in cultures ± microglia (*n* = 3 for no iron + RSL3; *n* = 4 biologically independent experiments for all other conditions). Two-way ANOVA, Sidak post hoc. *P* values are indicated in the graph. Error bars represent s.e.m. **f**, Lipid peroxidation in neurons (*n* = 3 for no iron + RSL3; *n* = 4 biologically independent experiments for all other conditions). Log-transformed, two-way ANOVA, Dunnett post hoc. *P* values are indicated in the graph. Error bars represent s.e.m. **g**, Correlation of neuronal surface area and neuronal lipid peroxidation (*n* = 33 wells examined over three biologically independent experiments). Two-tailed simple linear regression. **h**, Correlation of microglia number and neuronal surface area (*n* = 48 wells examined over four biologically independent experiments). Two-tailed simple linear regression. **i**, Correlation of microglia number and neuronal lipid peroxidation (*n* = 33 wells examined over three biologically independent experiments). Two-tailed simple linear regression. **j**,**k**, IL-8 (**j**) and IL-1β (**k**) production in supernatants from co-cultures ± microglia ± ferroptosis treatment (*n* = 4 biologically independent experiments). One-way ANOVA, Dunnett post hoc. *P* values are indicated in the graph. Error bars represent s.e.m. D, day.

Although iron sequestration is a key part of the microglial response, the FAS microglia also upregulated *CXCL8*/IL-8 mRNA (Fig. 2d and Extended Data Fig. 2i). IL-8, which serves as a chemoattractant, has been linked to neurodegenerative disorders, including ALS and PD^25^. To assess whether iron overload caused changes in cytokine secretion, we measured a panel of inflammatory cytokines in the cell culture supernatant at 18 hours after treatment. We identified a non-significant but 6.8-fold increase in IL-8 after iron treatment and a significant 7.8-fold increase in the iron + RSL3 condition of secreted IL-8 compared to control. Treatment with benzothiazine or lip-1 partially blocked increases in IL-8 production (Fig. 2j). At 18 hours, nine other cytokines were below the limit of detection. At a later timepoint (24 hours), IL-8 was still increased but no longer significant (*P* = 0.0763), and IL-1β was now detectable and significantly increased (Fig. 3j,k). Cultures lacking microglia had no detectable IL-8 (Fig. 3j). These results show that iron overload triggers a change in microglia cytokine production and release, which could contribute to the pathological environment in disease.

### Microglia exacerbate ferroptosis-driven neurodegeneration

The presence of iron-laden microglia in neurodegenerative disease and the shift in microglia cell state after iron overload raise the possibility that they could cause neuron death. To examine the role of FAS microglia in neurodegeneration, we established astrocyte–neuron co-cultures in the presence or absence of microglia (Fig. 3a). Remarkably, cultures without microglia had a marked delay and reduction of ferroptosis (Fig. 3b,c, Extended Data Fig. 3e and Supplementary Videos 1 and 2).

**Extended Data Fig. 4:**
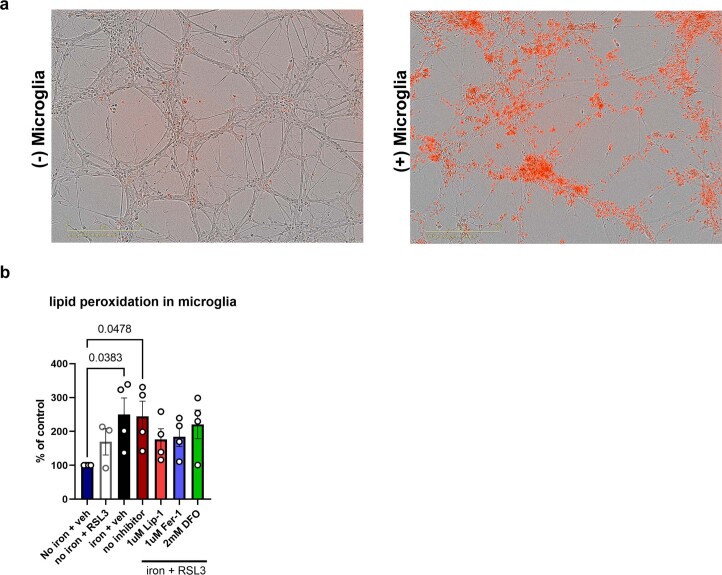
Microglia exhibit increased lipid peroxidation under ferroptotic conditions. **a**, Representative images of co-cultures ± microglia 24hrs post-ferroptosis treatment. Scale bar = 200 µm. **b**, lipid peroxidation in microglia 24hrs post treatment ± 1600µM ± 1 µM RSL3 ± ferroptosis inhibitor: 1 µM lip-1, 1 µM Fer-1, or 2 mM DFO. (n = 4 biologically independent experiments). One-way ANOVA, Dunnett post hoc. p-values are indicated in graph. Error bars represent SEM.

To directly assess whether this difference was due to neuronal loss, we performed immunocytochemistry and identified a significant loss of neuronal surface area only in co-cultures containing microglia (Fig. 3d,e). This is supported by live imaging data showing that the neuronal clusters in the culture failed to take up Draq7 in the absence of microglia (Extended Data Fig. 4a). We also found a significant increase in neuronal lipid peroxidation only when microglia were present (Fig. 3d,f). Notably, both the radical trapping antioxidants lip-1 and ferrostatin-1 (fer-1)^18^ and the iron chelator DFO rescued neuronal loss and partially rescued lipid peroxidation in the neurons and microglia (Fig. 3e,f and Extended Data Fig. 4b). Neuronal surface area negatively correlated with lipid peroxidation (Fig. 3g), and the number of surviving microglia after 24 hours ± ferroptosis treatment positively correlated with a reduction in neuronal surface area and negatively correlated with neuronal lipid peroxidation, suggesting a role for microglia death in neurotoxicity (Fig. 3h,i). Altogether, these results show that alterations in microglial phenotype and microglia death generate an environment that causes neuronal toxicity.

### Ferroptosis signature is enriched in neurodegenerative disease

**Fig. 4:**
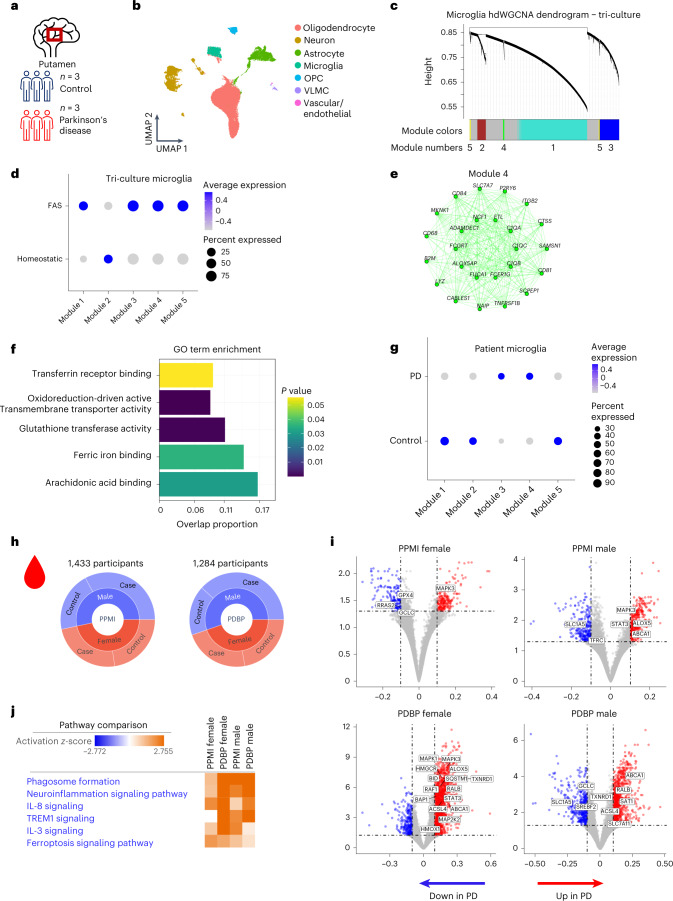
Detection of FAS signature in the microglia of patients with PD and a ferroptosis gene signature in the AMP PD patient blood samples. **a**, Brain region and sample size for nucSeq dataset. **b**, Unsupervised clustering and annotation of cell types identifies microglia cluster present in control and PD samples. **c**, Dendrogram identifying five hdWGCNA modules in FAS microglia. **d**, Dot plot for enrichment of modules in FAS and homeostatic microglia from tri-culture. **e**, Network plot showing top hub genes for module 4 (green). **f**, GO pathway analysis for module 4. Fisher exact test. **g**, Dot plot for enrichment of modules in PD and control microglia. **h**, Breakdown of participants in PPMI and PDBP cohorts by gender and disease status. **i**, Volcano plots for DEGs, −0.1 < log_2_ fold change < 0.1 and adjusted *P* < 0.05, between healthy controls and patients with PD stratified by gender and cohort. Wald test with DESeq2. **j**, IPA comparison analysis of DEGs (inclusion cutoffs: −0.1 < log_2_ fold change < 0.1 and adjusted *P* < 0.05 with male and female patients in PPMI and PDBP studies). The pathways were generated through the use of Qiagen IPA (https://digitalinsights.qiagen.com/IPA). VLMC, vascular lepotomeningeal cell.

There is strong evidence for a deleterious role for iron overload in many neurodegenerative diseases, including ALS, multiple sclerosis (MS) and PD^6,8,26–29^. However, we lack clear biomarkers to identify whether iron accumulation leads to ferroptosis in patients. We explored whether we could find enrichment of the ferroptosis signature from the iPSC tri-culture system in PD and ALS patient samples. We analyzed single-nucleus RNA sequencing (snRNA-seq) of postmortem putamen brain tissue from three patients with PD and three healthy controls (Fig. 4a)^30,31^. The putamen is connected to the substantia nigra and is a pathologically relevant area in the midbrain that is susceptible to iron accumulation in PD^32^. Progressive loss of spontaneous activity in the putamen also contributes to impaired task performance in patients with PD^33^.

**Extended Data Fig. 5:**
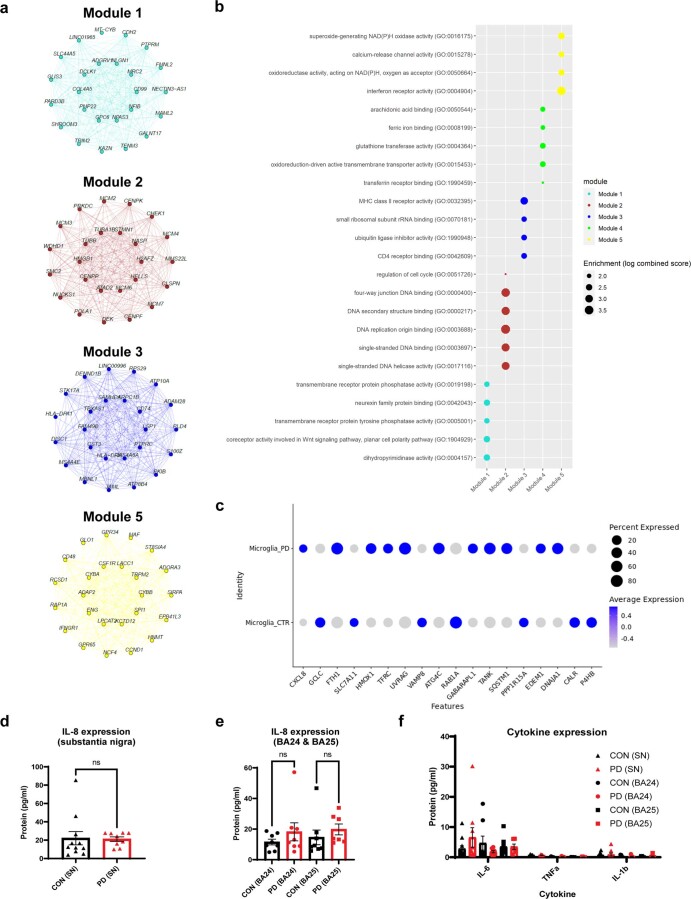
Tri-culture modules, pathway analysis, and IL-8 levels in control and Parkinson’s disease brain homogenates. **a**, Gene networks for modules 1,2,3, and 5. **b**, Select enriched pathways for tri-culture modules. **c**, Dotplot of expression for genes enriched in FAS microglia. **d**, IL-8 protein measured in human substantia nigra (SN) tissue by MSD (V-PLEX Proinflammatory Panel 1 Human Kit- K15049D). IL-8 protein was measured in control (CON) and Parkinson’s disease (PD) substantia nigra. (n = 10 con, n = 8 PD biologically independent samples). Two-tailed, unpaired t test. ns = non-significant. Error bars represent SEM. **e**, IL-8 protein measured in Brodmann’s Area 24 and 25 (BA24 & BA25). (n = 8 biologically independent samples). Two-way ANOVA, Sidak post hoc. ns = non-significant Error bars represent SEM. **f**, Expression of key cytokines including IL-6, TNFα, and IL-1β measured by MSD (V-PLEX Proinflammatory Panel 1 Human Kit- K15049D). Same samples measured as in (**a**) and (**b**) including SN, BA24 and BA25. No differences observed between CON and PD. Two-way ANOVA, Sidak post hoc. Error bars represent SEM.

**Extended Data Fig. 6:**
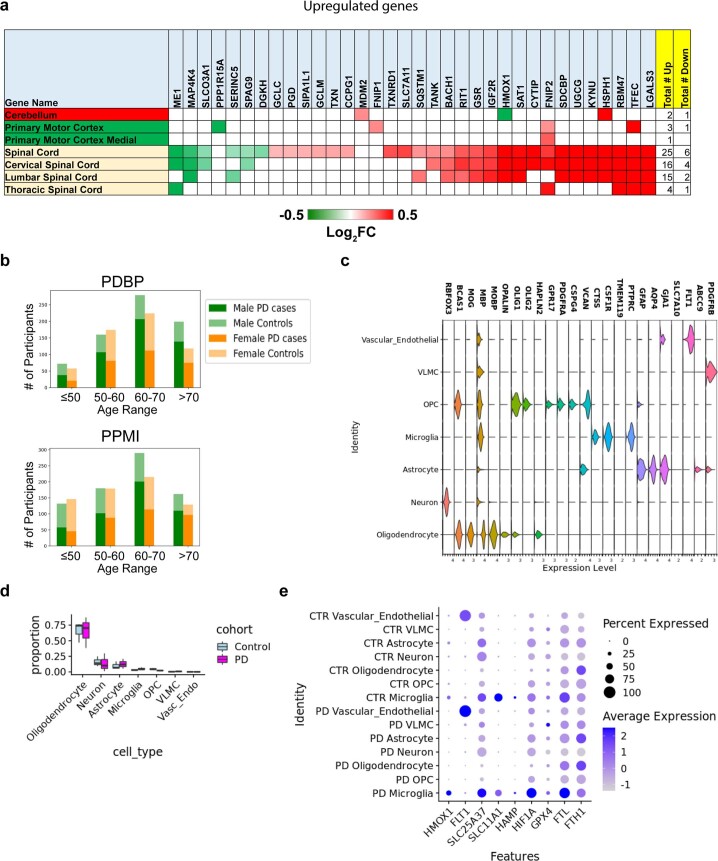
Ferroptosis-related gene expression in ALS patient spinal cord, information on AMP-PD cohorts, and cell type identification in PD single nucSeq and expression analysis of MS microglia iron-related signature in each cell type. **a**, Heatmap of upregulated genes in tri-culture ferroptotic microglia in ALS patients compared to control. Boxes shown in red or green are genes with P_adj_ < 0.05. The number of samples, area, and disease state analyzed are reported in Table S1. Statistical analysis performed with DESeq2. **b**, Breakdown of case, control, age, and gender for the PPMI and PDBP studies by the AMP-PD consortia. **c**, Violin plots for cell-type specific gene expression for endothelial cells, microglia, astrocytes, neurons, oligodendrocytes, and OPCs. **d**, proportions of each cell type from control and PD tissue. Minima = minimum value, maxima = maximum value, center = median, bounds of box = 25^th^ and 75^th^ percentile, bounds of whisker = minima and maxima. **e**, MS microglia iron-related signature was upregulated specifically in PD microglia compared to control and other cell types.

Unbiased clustering identified microglia, oligodendrocyte precursor cells (OPCs), oligodendrocytes, neurons, astrocytes and endothelial cells in the sequenced samples with similar numbers between PD and control (Fig. 4b and Extended Data Fig. 6c,d). We performed single-cell weighted gene co-expression network analysis (hdWGCNA) on the FAS microglia dataset to identify five gene modules associated with ferroptosis^34^ (Fig. 4c and Extended Data Fig. 5a). FAS microglia were significantly enriched for modules 3, 4 and 5, whereas homeostatic microglia were significantly enriched for module 2 (Fig. 4d). Pathway analysis was run on all five modules (Extended Data Fig. 5a,b). Module 4 was enriched for genes associated with iron dysregulation, glutathione transferase activity, ferric iron binding and arachidonic acid binding (Fig. 4d–f). This iron-related module 4, as well as module 3, were significantly enriched in PD microglia versus healthy controls (Fig. 4g). Additionally, many of the genes upregulated in FAS microglia were also upregulated in the PD microglia, including *CXCL8*, *FTH1*, *ATG4C* and *EDEM1* (Fig. 2d and Extended Data Fig. 5c), similar to a recent signature uncovered in iron-laden microglia in progressive MS lesions^8,9^ (Extended Data Fig. 6e). To examine whether changes in *CXCL8* transcription could also be detected at the protein level, we measured IL-8 and other inflammatory cytokines (IL-6, TNF-α and IL-1β) in the substantia nigra and Brodmann’s area 24 and 25 in a separate cohort of patients with PD but found no differences compared to control samples (Extended Data Fig. 5d–f). This could be due to a lack of sensitivity in whole tissue measurements or could be related to the cohort of patients used for these measurements. Altogether, we identify a core iron dysregulation module that is upregulated and enriched in both FAS microglia and PD microglia, further suggesting that iron dyshomeostasis and ferroptosis are active in human disease.

In ALS, iron accumulation has been reported in the deep cortical layers as well as in microglia in the motor cortex^6^. We compared gene expression changes between patients with ALS and case controls from the Target ALS consortia (Supplementary Table 1) for 50 select genes that were upregulated in the FAS microglia. We found significant enrichment of 34/50 genes especially in the spinal cord of patients, including *SLC7A11* and *GCLM*, which were upregulated across all cell types in the tri-culture. There was also dysregulation in the motor cortex, frontal cortex, occipital cortex and cerebellum (Extended Data Fig. 6a). These results show a strong, shared ferroptosis-associated signature in microglia specifically in disease-afflicted areas across multiple neurodegenerative disorders, including MS, PD and ALS.

Our data show that ferroptosis-related pathways, including those associated with FAS microglia, are upregulated in postmortem brain tissue, but, to examine whether this signature could be detected in living patients, we analyzed blood RNA-seq data from a large cohort of healthy and PD participants enrolled in cohort studies of the Accelerating Medicines Partnership in Parkinson’s Disease (AMP PD), the Parkinson’s Progression Markers Initiative (PPMI) (*n* = 1,433; 816 PD cases and 617 controls) and the Parkinson’s Disease Biomarkers Program (PDBP) (*n* = 1,284; 780 PD cases and 504 controls) (Fig. 4h and Extended Data Fig. 6b). Differential gene expression analysis between PD cases and healthy controls uncovered significantly upregulated and downregulated ferroptosis-related genes, many of which were found in our tri-culture FAS microglia signature, including *GCLC*, *GPX4*, *HMOX1*, *SQSTM1* and *SLC7A11* (Fig. 4i). Indeed, ferroptosis and IL-8 signaling were among the top dysregulated pathways identified using ingenuity pathway analysis (IPA)^35^ (Fig. 4j). These findings show that a ferroptotic gene signature is present in blood samples derived from patients with PD and that iron dysregulation may occur systemically in patients. Furthermore, these findings show that blood samples could potentially be used as a peripheral biomarker for ferroptosis patient identification and stratification in PD and other neurodegenerative diseases.

### CRISPR screen identifies microglia ferroptosis regulators

**Extended Data Fig. 7:**
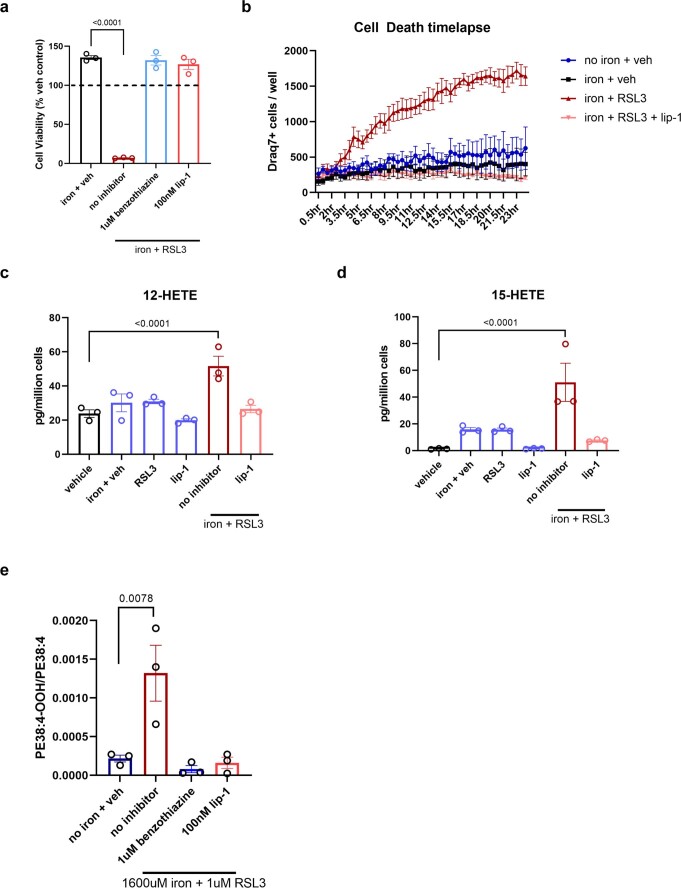
Lipidomic analysis reveals distinct ferroptotic signature in microglia. **a**, Ferroptosis induction (800 µM iron + 1 µM RSL3) ± inhibitors (1 uM benzothiazine/100 nM lip-1) in human immortalized microglia cell line. (n = 3 biologically independent experiments). One-way ANOVA, Dunnett post hoc. p-values are indicated in graph. Error bars represent SEM. **b**, Draq7+ death kinetics of human immortalized microglia exposed to 400 µM iron + 1 µM RSl3 ± 100 nM lip-1. (n = 3 wells). Error bars represent SEM. **c** and **d**, Lipidomic analysis shows increased free 12-HETE and 15-HETE in immortalized human microglia 2 hr post-treatment. (n = 3 biologically independent experiments). One-way ANOVA, Sidak post hoc. p-values are indicated in graph. Error bars represent SEM. **e**, Production of 1-SA-2–15-HpETE-PE ± or 1 µM benzothiazine or 100 nM lip-1. (n = 3 biologically independent experiments). One-way ANOVA, Dunnett post hoc. p-values are indicated in graph. Error bars represent SEM.

Our data show that microglia play a key role in ferroptosis-driven neurodegeneration. Because susceptibility to ferroptosis varies among cell types^19,36^, we sought to determine if there are unique regulators of ferroptosis in microglia. Genome-wide screens for ferroptosis regulators have been performed in several cancer cell lines^37–41^ but never in microglia or other brain cell types. We performed a positive selection genome-wide CRISPR screen using Cas9-expressing immortalized human microglia to allow for the scalability needed for a genome-wide analysis. This microglia line is derived from the adult human brain and is susceptible to lipid peroxidation and ferroptosis^8,11^ (Extended Data Fig. 7a,b). Lipidomic analysis revealed increased production of ferroptosis-associated lipid and phospholipid peroxides after exposure to iron + RSL3 (Extended Data Fig. 7c–e).

**Fig. 5:**
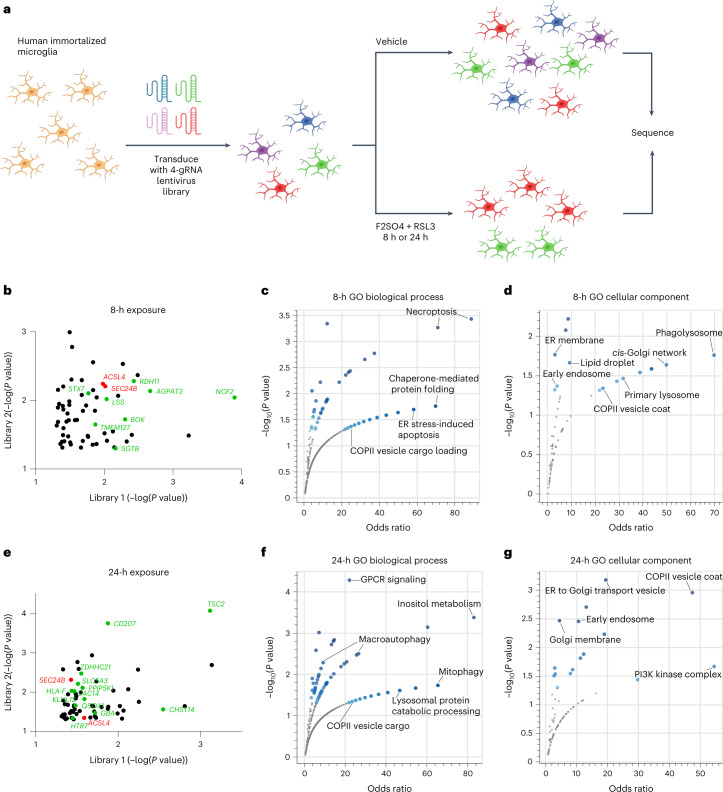
Genome-wide CRISPR screen identifies *SEC24B* as a novel regulator of ferroptosis in microglia. **a**, Schema for positive selection genome-wide CRISPR screen in Cas9-expressing human immortalized microglia. Illustrations were created with BioRender. **b**,**e**, Scatter plot of overlapping hits from each viral pool after 8-hour exposure (**b**) or 24-hour exposure (**e**). Genes highlighted in green were identified in at least one of the GO-associated pathways. Statistical analysis was performed by MAGeCK. *ACSL4* and *SEC24B* are highlighted in red as the two genes identified across both timepoints and viral pools. **c**,**d**, Top associated pathways determined by GO biological process (**c**) and GO cellular component (**d**) for 8-hour exposure. **f**,**g**, Top associated pathways determined by GO biological process (**f**) and GO cellular component (**g**) for 24-hour exposure. Fisher exact test was performed for all pathway analyses.

**Extended Data Fig. 8:**
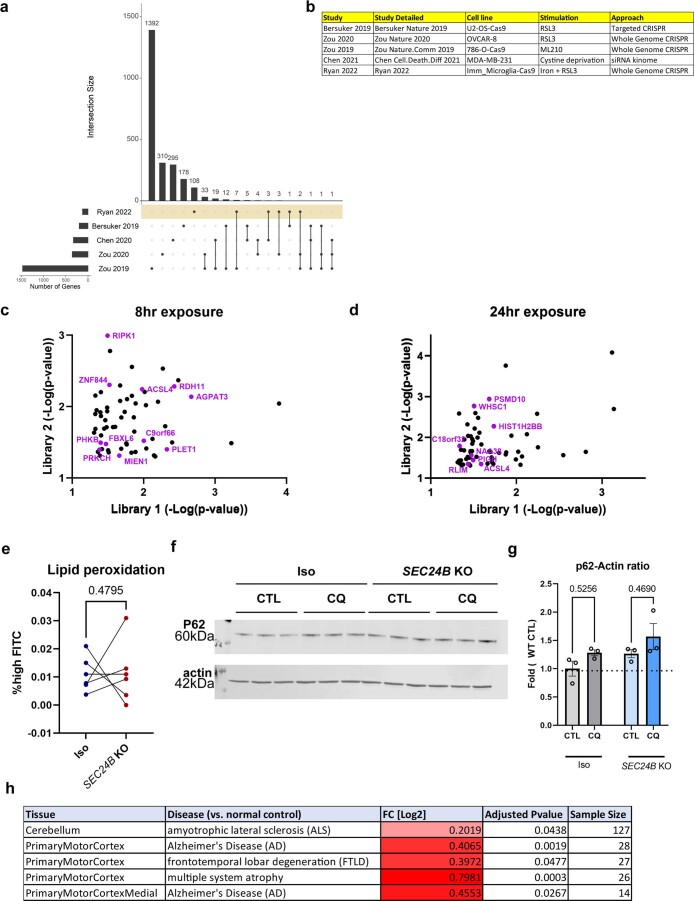
CRISPR Screen additional information *SEC24B* disease association and flow cytometry. **a**, intersection of hits from various published CRISPR and siRNA screens. **b**, Details on cell type, treatment, and screen approach for various published works. **C** and **d**, Scatter plots of overlapping hits from the two viral pools in 8 hr and 24 hr treatments. Statistical analysis performed by MAGeCK. Genes highlighted in purple were identified in previously published ferroptosis CRISPR screens. **e**, Lipid peroxidation as defined by percent of FITC^bright^ PE+ cells in isogenic and *SEC24B* KO lines when treated with vehicle (n = 6 biologically independent experiments). One-tailed paired t test. p-values are indicated in graph. **f**, Western blot for P62 in isogenic and *SEC24B* KO HAP1 cell lines serum-starved and treated with vehicle or 100 µM chloroquine (CQ) for 6hrs. **g**, Quantification of western from **f** (n = 3 biologically independent experiments). One-way ANOVA, tukey post-hoc. p-values are indicated in graph. Error bars represent SEM. **h**, Expression of *SEC24B* in multiple neurodegenerative diseases.

We used a 76,612-guide library targeting 19,114 genes with four guides per gene. Cells were transduced with two separately generated viral pools and treated with vehicle or iron + RSL3 for 8 hours or 24 hours for less and more stringent selection (Fig. 5a). We identified 61 hits from the 8-hour exposure (Fig. 5b) and 65 hits from the 24-hour exposure (Fig. 5e). From the 124 unique hits, 18 hits were previously identified in other ferroptosis screens (Extended Data Fig. 8a–d)^39,41–45^. These data suggest that there is a core network of ferroptosis regulators in multiple cell types, but that many regulators could be cell type specific.

Gene Ontology (GO) analysis for biological processes and cellular components uncovered genes associated with ER stress and transport and ER-Golgi vesicle transport at both timepoints (Fig. 5c,d,f,g). Interestingly, these pathways were also enriched in FAS microglia (Fig. 2d,e). *ACSL4*, a well-established ferroptosis regulator^37–39^, and *SEC24B*, a novel factor identified in this study, were the only two genes present in both timepoints and libraries (Fig. 5b,e). *SEC24B* is a regulator of COPII-mediated protein trafficking from the ER to the Golgi apparatus^46^. The COPII system was enriched in both GO categories at both timepoints, and other COPII genes, including *KLHL12* and *HLA-F*, also contributed to ferroptosis susceptibility in microglia. Autophagy, lysosomal, and endosomal pathways were also enriched (Fig. 5c,d,f,g). *SEC24B* has been implicated in autophagosome formation in the ER-Golgi intermediate compartment (ERGIC), and autophagy has been implicated in ferroptosis through ferritinophagy^47^. This association of ER-Golgi and autophagy-related pathways and the connection to *SEC24B* led us to investigate the potential mechanism underlying *SEC24B* regulation of ferroptosis.

### *SEC24B* knockout prevents ferroptosis in microglia

**Fig. 6:**
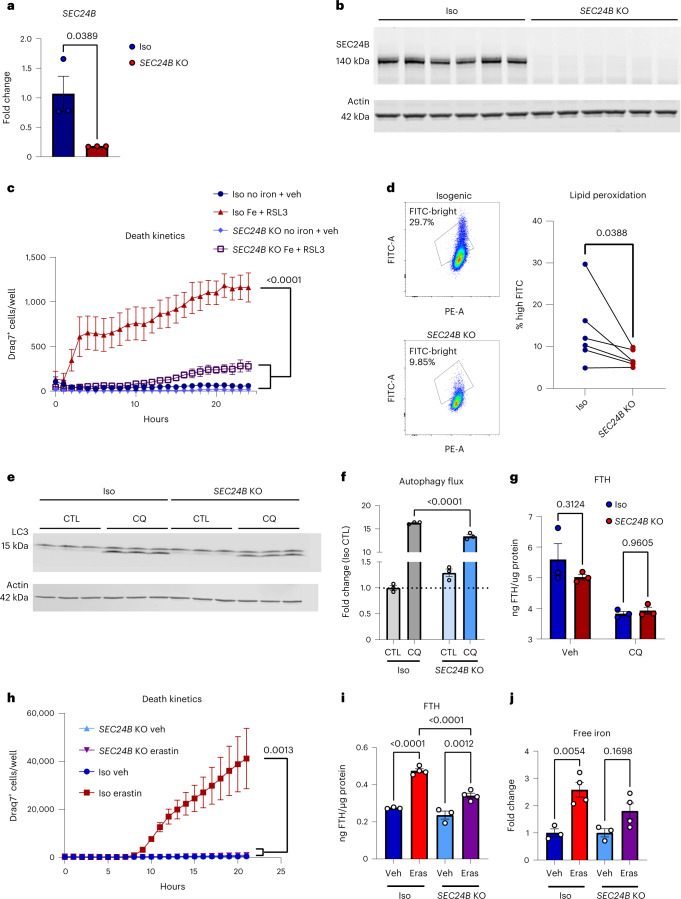
SEC24B is a potent regulator of ferroptosis. **a**, qRT–PCR analysis showing markedly reduced expression of *SEC24B* in KO line (*n* = 3). Unpaired *t*-test. *P* values are indicated in the graph. Error bars represent s.e.m. **b**, Western blot showing absence of SEC24B protein in KO line (*n* = 6 biologically independent samples). **c**, Death kinetics in *SEC24B* KO HAP1 cell line and isogenic control treated with vehicle (no iron + vehicle) or 1,600 µM iron + 1 µM RSL3 (iron + RSL3) (*n* = 3 biologically independent experiments). AUC, one-way ANOVA, Dunnett post hoc. *P* values are indicated in the graph. Error bars represent s.e.m. **d**, Lipid peroxidation in *SEC24B* KO HAP1 cell line and isogenic control 2–4 hours after ferroptosis treatment (*n* = 6 biologically independent experiments). One-tailed, paired *t*-test. *P* values are indicated in the graph. **e**,**f**, Western blot (**e**) and quantification of autophagy flux (ratio of LC3-II:LC3-I) (**f**) in *SEC24B* KO HAP1 cell line and isogenic control 6 hours after serum starvation and 100 µM chloroquine (CQ) treatment (*n* = 3 biologically independent experiments). One-way ANOVA, Tukey post hoc. *P* values are indicated in the graph. Error bars represent s.e.m. **g**, FTH ELISA in *SEC24B* KO HAP1 cell line and isogenic control 6 hours after serum starvation and 100 µM CQ treatment (*n* = 3 biologically independent experiments). Two-way ANOVA, Sidak post hoc. NS, not significant. Error bars represent s.e.m. **h**, Death kinetics in *SEC24B* KO HAP1 cell line and isogenic control treated with vehicle or 10 µM erastin (*n* = 3 biologically independent experiments). AUC, one-way ANOVA, Dunnett post hoc. *P* values are indicated in the graph. Error bars represent s.e.m. **i**, FTH ELISA in *SEC24B* KO HAP1 cell line and isogenic control 24 hours after 10 µM erastin (Eras) treatment (*n* = 3 for vehicle and *n* = 4 for Eras biologically independent experiments). One-way ANOVA, Tukey post hoc. *P* values are indicated in the graph. Error bars represent s.e.m. **j**, Free, reduced iron quantification in *SEC24B* KO HAP1 cell line and isogenic control 24 hours after 10 µM Eras treatment (*n* = 3 for vehicle and *n* = 4 for Eras biologically independent experiments). One-way ANOVA, Tukey post hoc. *P* values are indicated in the graph. Error bars represent s.e.m. CTL, control; veh, vehicle.

To explore the role of SEC24B as a regulator of ferroptosis, we assessed ferroptosis susceptibility in an *SEC24B* knockout (KO) myeloid cell line. *SEC24B* KO was confirmed by quantitative PCR with reverse transcription (qRT–PCR) and an absence of protein by western blot (Fig. 6a,b). To assess ferroptosis susceptibility, cells were exposed to iron + RSL3, and cell death was measured by Draq7 uptake over 24 hours. The *SEC24B* KO cells were highly resistant to ferroptosis with a fourfold reduction in ferroptosis as compared to the control isogenic line (Fig. 6c). *SEC24B* KO cells also had significantly reduced lipid peroxidation after 2–4-hour exposure to ferroptotic stimuli, with no basal difference in lipid peroxidation (Fig. 6d and Extended Data Fig. 8e). These results demonstrate that *SEC24B* is a strong regulator of ferroptosis induction in microglia and macrophages.

SEC24B plays a role in autophagic flux by binding SEC24A and SEC23B and localizing to the ERGIC in response to starvation. Once there, the complex can help produce autophagosomes^48^. Interestingly, SEC24B-COPII localization to the ERGIC is dependent on PI3K, an enriched pathway in the CRISPR screen (Fig. 5f,g)^48,49^. To explore a potential role of *SEC24B* and autophagy in ferroptosis susceptibility, we exposed *SEC24B* KO and isogenic control HAP1 cells to chloroquine, an inhibitor of autophagy, and identified a significant impairment in autophagic flux in the *SEC24B* KO cells with reduced LC3 I/II ratio (Fig. 6e,f). P62 levels were increased in the *SEC24B* KO but the changes were not significant (Extended Data Fig. 8f,g). Altered autophagic flux could contribute to ferroptosis susceptibility in several different ways, but one type of autophagy called ferritinophagy causes ferroptosis through ferritin degradation and release of free, labile iron^50^. To explore whether SEC24B also regulates ferritinophagy, we measured ferritin levels under autophagic conditions but found that ferritin levels were not changed (Fig. 6g). To use a more ferroptosis-relevant stimuli, we exposed the cells to erastin, another strong ferroptosis inducer that has been shown to drive altered iron homeostasis and ferritinophagy^10,23,51^. *SEC24B* KO cells were strongly resistant to erastin-induced ferroptosis (Fig. 6h), and erastin treatment led to significant increases in ferritin and free, labile iron in the isogenic control, but the increase in ferritin was reduced, and there was no difference in labile iron in the *SEC24B* KO cells (Fig. 6i,j). These findings suggest that, although loss of *SEC24B* leads to impaired autophagic flux, it likely does not regulate ferroptosis through ferritinophagy. However, these results show that *SEC24B* does alter the labile iron pool, a key upstream trigger of the ferroptosis cascade, which could explain the protective effect of *SEC24B* KO.

Although *SEC24B* has not been identified before as a regulator of ferroptosis, it is significantly upregulated in multiple neurodegenerative diseases, including ALS, multiple system atrophy, frontotemporal lobar degeneration and Alzheimer’s disease (Extended Data Fig. 8h). These diseases have been associated with iron dysregulation and ferroptosis^2,52–54^. Upregulation of *SEC24B* could be contributing to ferroptosis induction and iron dysregulation in these diseases. However, further experiments are necessary to fully understand the role of *SEC24B* in ferroptosis-mediated neurodegeneration.

### Compounds targeting CRISPR hits inhibit ferroptosis

**Fig. 7:**
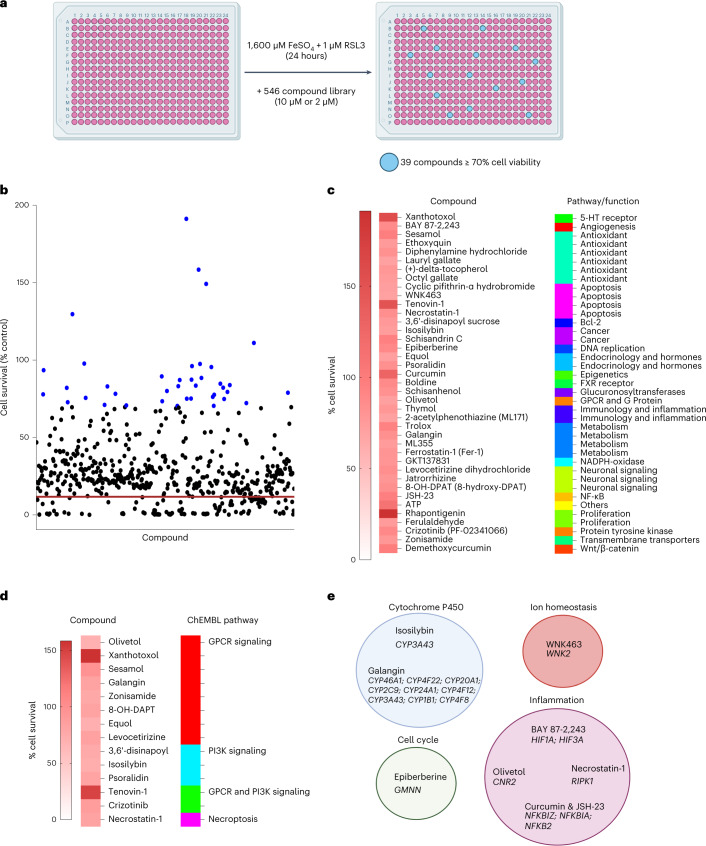
Ferroptosis pharmacological inhibitors revealed in small-molecule screen. **a**, Schema and results for pharmacological compound screen in which 546 compounds were tested for their ability to block ferroptosis in an immortalized microglia cell line. Illustrations were created with BioRender. **b**, Thirty-nine of the 546 compounds rescued cell viability to at least 70% of the vehicle control (blue dots), and five rescued to at least 100%. Three technical replicates per compound. Red line indicates average cell survival with ferroptosis induction and no compound. **c**, Heat map of percent cell survival for the 39 hits and the Selleck-associated pathway or function. **d**, Heat map of percent cell survival for compounds predicted to interact with GPCR signaling, PI3K signaling or necroptosis by ChEMBL. **e**, Correlation between the targets of pharmacological hits and genes identified in the CRISPR screen that are associated with the targets, separated by general pathway/function. Illustrations were created with BioRender.

Given a potential role for microglia ferroptosis in neurodegeneration, we sought to identify compounds that inhibit ferroptosis in microglia. To do this, we performed a targeted small-molecule screen with a commercially available, ferroptosis-related library that consists of 546 compounds, including some that are already in clinical use^55,56^. The human Cas9-expressing microglia cell line was exposed to iron + RSL3 and co-treated with the compounds at 10 µM (535/546) or 2 µM (11/546). Inhibition of ferroptosis was noted for any compound that led to ≥70% cell viability compared to vehicle control. Of the 546 compounds, we found 39 compounds that inhibited ferroptosis in microglia (Fig. 7a–c and Supplementary Table 2). The 39 hits were analyzed on the bioactivity database ChEMBL^57^ to determine on which pathways they are predicted to act. This analysis showed that PI3K signaling, GPCR signaling and necroptosis pathways were enriched in the screen hits (Fig. 7d). Correspondingly, these pathways were also identified in the CRISPR screen (Fig. 5c,d,f,g). Several other CRISPR hits were related to targets of the compound hits. For example, two cytochrome P450 inhibitors (galangin and isosilybin) were hits in the chemical screen, and many cytochrome P450-related genes, including *CYP3A43*, *CYP46A1* and *CYP1B1*, were hits in the CRISPR screen. There were also multiple inflammation-related genes, such as *HIF1A*, *CNR2* and *NFKBIZ*, from the CRISPR screen that are targeted by BAY 87–2243, olivetol and curcumin and JSH-23, respectively (Fig. 7e). Thus, the pharmacological screen identified an array of compounds and validated multiple pathways and genes identified in the CRISPR screen.

Several chemical screen hits corroborated previously identified regulators of ferroptosis. Cytochrome P450 oxidoreductase, which uses NADPH, has been recently identified in a separate genome-wide screen^39^. Galangin targets cytochrome P450, and ML171 and GKT137831 both target NADPH-oxidase (Supplementary Table 2). p53 has also recently been identified as an alternative pathway for ferroptosis induction^58^. In the screen, p53 inhibitors cyclic pifithrin-α hydrobromide and tenovin-1 were effective at preventing ferroptosis. Lastly, HIF-1α was upregulated in the ferroptotic microglia in the tri-culture, and BAY 87–2243 is a HIF-1α inhibitor in phase 1 clinical trials for cancer. These results identified new genetic regulators of ferroptosis in microglia and validated pharmacological compounds, some already in clinical use, which can be used for the treatment of ferroptosis-related neurodegenerative diseases.

## Discussion

Iron accumulation in microglia in neurodegenerative disease has been well described, but the consequences of iron on microglia state and function are not well understood. Here we identify microglia as a major player in a cellular ferroptotic cascade, assuming a distinct neurotoxic state that contributes to neurodegeneration. In the tri-culture model, the microglia are the first to develop a ferroptotic response and may act as an initiator of ferroptosis in neurons through the production of inflammatory factors, including IL-8 or Il-1β^25,59^. In fact, others have also shown that microglia release IL-6 in the presence of α-synuclein, which leads to increased iron uptake and cell death in neurons^60^, supporting the idea that FAS-derived factors induce ferroptosis-dependent neurodegeneration. We use a well-established^10^ but artificial method to induce ferroptosis via GPX4 inhibition with RSL3. In disease, however, ferroptosis likely occurs more slowly, over a greater period of time, via accumulation of iron, loss of glutathione and buildup of toxic lipid peroxides,^2,61,62^. Although it is still difficult to model these slow, disease-related processes, these differences should be considered when interpreting results from iron-induced and RSL3-induced death in the in vitro tri-culture model.

We and others previously identified a transcriptomic signature of iron-laden microglia in progressive MS^8,9^, but whether this signature was triggered by ferroptosis or iron overload was unclear. To address this issue, we induced ferroptosis in the human iPSC tri-culture and used this signature to explore enrichment in neurodegenerative disease. Using scRNA-seq, we found a unique ferroptotic gene signature in our iPSC tri-culture microglia that was dysregulated in microglia from PD patient putamen and in ALS patient spinal cord. This supports the utility of our human iPSC-derived tri-culture system to identify disease-relevant ferroptosis signatures that could be translated to human disease. This was previously very difficult due to a lack of specific ferroptosis markers^63^, and this signature could help determine when and where ferroptosis occurs and lead to identification of biomarkers that could help stratify patients in the clinic. Although we did not identify an increase in IL-8 or other cytokines in patient brain tissue, this may be due to a relatively small sample size and/or lack of sensitivity in whole tissue measurements, because only a small subset of microglia upregulate IL-8, or that these patients had limited neuroinflammation. There are reports of altered IL-8 levels in plasma and cerebrospinal fluid (CSF) from other cohorts of patients with PD^64,65^, but we think that the utility of IL-8 as a ferroptosis-specific biomarker is still limited because IL-8 can be triggered by other stimuli. However, an iron-triggered cytokine response has not been previously appreciated and could highlight a range of factors that are released from FAS microglia that drive neuroinflammation and neurodegeneration. Future studies with larger cohorts of patients with PD will be needed to assess whether IL-8 or other FAS-related factors are elevated at the protein level in PD microglia. We uncovered signs of ferroptosis in two separate tissues from patients with PD, blood and brain, and future examination of ferroptosis markers in patient CSF could provide a clinically relevant biofluid to bridge the nervous system and periphery. It will be key to determine whether the signal in the blood reflects a response to central nervous system (CNS) pathology in PD or a systemic dysregulation of iron pathways and immune cells in blood. Tracking putative ferroptosis biomarkers in CSF will also be critical for the goal of patient identification and could be paired with iron bioimaging.

Surprisingly, we found that microglia drive ferroptosis-dependent neurodegeneration. Co-cultures without microglia had a significant delay and magnitude of ferroptosis induction in the neurons. In disease, microglial uptake of iron may initially be protective, but, as the cells succumb to ferroptosis, they enter a neurotoxic cell state that leads to damage. In support of this mechanism, in our tri-culture system, neurodegeneration occurs after the microglia die en masse. These findings also strongly suggest that the dying microglia release factors into the milieu that exacerbate neuronal death. We identify IL-8 and IL-1β as two potential cytokines that could contribute to the neuroinflammatory environment after iron overload. However, these cytokines are triggered by many different stimuli, and it is likely that multiple factors are involved in iron-driven and microglia-driven neurotoxicity. Further studies are necessary to determine which factors are causing neuron death and uncover ways to specifically target FAS microglia.

Before this study, regulation of ferroptosis in microglia was not well understood. Using a genome-wide CRISPR screen, we discovered that, in addition to the well-described regulator *ACSL4*, *SEC24B* also strongly regulates ferroptosis in microglia. *SEC24B* has never previously been implicated in ferroptosis and is a COPII vesicle protein involved in cargo packaging and trafficking from the ER to the Golgi apparatus, particularly for secretory proteins^46^. COPII, ER, Golgi apparatus and lysosomal-related and autophagy-related pathways were consistently associated with the CRISPR screen hits, and autophagy and ER protein processing were major affected pathways in the FAS microglia. Although the *SEC24B* KO cells had lower autophagic flux, there was no impact on ferritin degradation or ferritinophagy^47^. Our data suggest that SEC24B impacts iron homeostasis by regulating the labile iron pool, through mechanisms that could include altered export of iron regulatory proteins or transporters or though other indirect pathways. These results suggest that protection in *SEC24B* KO cells is upstream and provides protection, in part, by reduced iron exposure.

Despite the fact that ferroptosis has been implicated in many disorders, an effective therapeutic to mitigate ferroptosis has not been approved for patients^66^. Iron chelators are one potential approach, but many have unknown or poor blood–brain barrier permeability and may disrupt homeostatic redox functions^67^. However, existing preclinical studies using lipid peroxidation inhibitors, such as lip-1, and the data presented in this study provide a strong rationale for developing ferroptosis-targeting therapeutics. Several compounds targeting lipid peroxidation and oxidative stress are in clinical trials, including the vitamin E derivative vatiquinone, deuterated linoleic acid and activators of the antioxidant NRF2 pathway. We screened a commercially available library of compounds and identified several candidates that could prevent microglia ferroptosis. This included the HIF-1α inhibitor BAY 87–2243, which is in phase 1 clinical trials for cancer, as well as the FDA-approved antioxidant octyl gallate. Altogether, this work identifies a key role for iron-laden microglia in neurodegenerative disease and suggests that these cells are not merely a byproduct of underlying disease processes but could also be contributing to disease progression and neurodegeneration.

## Methods

### Tri-culture assembly

On day 0, 96-well plates (PerkinElmer, 6055302, or Corning, 3595) were coated with Matrigel (Corning, 354277) (diluted in appropriate amount of DMEM (Life Technologies, 11330057) solution). Then, 200 µl of sterile PBS (Thermo Fisher Scientific, 20012-027) was added to any unused wells. iAstrocytes (Fujifilm, ASC-100-020-001-PT) were thawed and plated at 1.5 × 10^4^ per well in Fuji-designated astrocyte media (200 µl per well) (DMEM/F12, HEPES (Life Technologies 11330057) + 2% heat-inactivated FBS Certified One Shot (Gibco, A38400-01) + 1× N-2 supplement and 100× (Gibco, 17502-048)). On day 1, 3.5 × 10^4^ iCell motor neurons (Fujifilm, C1048) were added per well. Motor neurons were thawed and resuspended at 3.5 × 10^4^ cells per 200 µl in complete Fuji motor neuron media (100 ml of iCell Neural Base Medium 1 (Fujifilm, M1010) + 2 ml of iCell Neural Supplement A (Fujifilm, M1032) + 1 ml of iCell Nervous System Supplement (Fujifilm, M1031)). Fuji astrocyte media was fully aspirated, and 200 µl of motor neuron cell suspension was added to each well. On day 3, a 75% media exchange (150 µl) was performed in all wells with NB/B27 + media (B-27 Plus Neuronal System Kit (Life Technologies, A3653401)). On day 5, iCell Microglia (Fujifilm, C1110) were plated at 1 × 10^4^ per well in NB/B27 + iMg growth factors (B-27 Plus Neuronal System Kit (Life Technologies, A3653401) + 25 ng ml^−1^ of M-CSF (PeproTech, 300-25) + 100 ng ml^−1^ of IL-34 (PeproTech, 200-34) + 50 ng ml^−1^ of TGF-β1 (PeproTech, 100-21)). iMicroglia were thawed and resuspended in NB/B27 + iMg growth factors at 1 × 10^4^ cells per 100 µl. Then, 100 µl of media was removed from each well, and 100 µl of iMicroglia cell suspension was added. Half media exchanges were performed on day 7, day 9, day 11 and day 14. All treatments were added on day 15.

### Tri-culture ferroptosis treatments

2× solutions were made for all tri-culture treatments. Half media exchanges with 2× treatments were performed. Final concentrations were 1:1,000 DMSO (Sigma-Aldrich, D2650), 1,600 µM FeSO_4_ (Sigma-Aldrich, F8633), 1 µM RSL3 (Sigma-Aldrich, SML2234), 10 µM 4-methyl-2-(4-methylpiperazinyl)pyrimido[4,5-*b*]benzothiazine (Cayman Chemical, 10010468), 1 µM lip-1 (Sigma-Aldrich, SML1414), 2 mM DFO (Sigma-Aldrich, D9533) and 1 µM fer-1 (Sigma-Aldrich, SML0583). Cultures were treated for 6–42 hours depending on experiment. All inhibitors were added as co-treatments.

### scRNA-seq cell preparation

Six hours after treatment, cells were washed 2× with PBS. Cells were then treated with 0.25% trypsin + EDTA (Sigma-Aldrich, T4049) + transcription/translation inhibitors (5 µg ml^−1^ of actinomycin-D (Sigma-Aldrich, A1410) + 10 µM triplotide (Sigma-Aldrich, T3652) + 27.1 µg ml^−1^ of anisomycin (Sigma-Aldrich, A9789)) for 6 minutes at 37 °C in a 5% CO_2_ incubator. Then, 1 volume of PBS + 2% FBS (Gibco, A38400-01) + transcription/translation inhibitors + 1:100 DNAse was added to quench. Cells were combined from two wells and put through 40-µm cell strainers (BD Falcon, 352235). Strained cell suspension was placed in a 1.7-ml Eppendorf tube on ice. Cells were counted by hemocytometer. Cell suspensions were spun at 4 °C for 5 minutes at 528 *g*. Supernatant was removed, and cells were resuspended at 1,000 cells per microliter in ice-cold PBS.

### scRNA-seq

Cells were loaded onto the 10x Genomics Next GEM Single Cell 3′ v3.1 sample chip and run following the manufacturerʼs protocols. All 15 samples were processed in parallel and were amplified 11 cycles during the initial cDNA amplification and 12 cycles for the sample index PCR. The samples were sequenced on an Illumina NovaSeq 6000 to an average depth of approximately 40,000 reads per cell. Sequencing files were processed using the Cell Ranger 5 pipeline and the GRCh38-2020-A human reference genome.

### scRNA-seq analysis

Samples were analyzed using Seurat 4.0.6 (ref. ^68^) in R version 4.0.3. Samples were filtered based on the overall distribution of unique molecular identifiers (UMIs) and gene counts per cell to limit the inclusion of low-quality cells and doublets. Cells with fewer than 4,000 genes per feature and 10,000 UMIs and more than 10,000 genes, 80,000 UMIs and 20% mitochondrial genes were removed from analysis. After filtering, the variable features were selected using the ‘vst’ method and using 2,000 features. RunICA was performed for 100 independent components (ICs), and then the FindNeighbors function was run using 50 ICs to build the *k*-nearest neighbor (KNN) graph. Cluster resolution was set to ‘0.4’, and the uniform manifold approximation and projection (UMAP) was generated using the RunUMAP function and using 50 ICs. Cell types were assigned by identifying genes unique to each cluster and through cross-referencing to known markers of each cell type and existing published datasets. UMAP plots and gene expression plots were generated using built-in Seurat/ggplot2 plotting functions or scCustomize version 0.7.0 unless otherwise described. Cluster identities were determined by calculating enriched markers using the FindAllMarkers function in Seurat.

To calculate the percent of cells per cluster from each sample, the number of cells from each sample in a given cluster was calculated and normalized to the number of cells per sample. These values were then normalized to the other replicate samples. Significantly enriched samples were identified using a two-way ANOVA with Tukey post hoc test, and *P* values are reported in each figure.

### Pseudo-bulk analysis

For the unbiased analysis of ferroptosis-related genes, pseudo-bulk gene matrices were generated for each sample by calculating the row sums for each gene. The samples were then all combined into a single gene counts matrix and analyzed using DESeq2 with the treatment condition as the variable of interest^69^. Genes with count sums equaling 0 were removed from the analysis. Statistically significant genes (adjusted *P* < 0.05) in the iron + RSL3 versus vehicle groups were identified and then used for making plots and for downstream analysis. The top 100 upregulated genes were plotted using the Pheatmap package version 1.0.12 in R using unbiased hierarchical clustering.

### UCell analysis

To determine the cell type enrichment of the ferroptosis signature generated from the pseudo-bulk analysis, UCell package version 1.1.0 was used (Andreatta and Carmona^21^). The UCell signature score is based on the Mann–Whitney *U* statistic and is agnostic to dataset cell type composition when assigning scores. The top 50 upregulated genes from the pseudo-bulk analysis were used to calculate the UCell score for each cell in the dataset. UCell score violin plots were grouped by cell subtype (microglia 1–2, neuron 1–3 and astrocyte 1–12) and plotted using Seurat/ggplot2.

### Microglia subclustering analysis

Microglia were subset from the larger dataset based on cell type definitions presented in Fig. 1 and reanalyzed using Seurat. The variable features were selected using the ‘vst’ method and using 2,000 features. RunICA was performed for 30 ICs, and then the FindNeighbors function was run using 18 ICs to build the KNN graph. Cluster resolution was set to ‘0.4’, and the UMAP was generated using the RunUMAP function and using 18 ICs. UMAP plots and gene expression plots were generated using built-in Seurat/ggplot2 plotting functions unless otherwise described. FAS-enriched genes for Fig. 2 were identified using the FindMarkers function in Seurat and using DESeq2 as the differential expression test.

### Cytokine analysis

Supernatants from tri-cultures were run on Proinflammatory Panel 1 (human) (Meso Scale Discovery, K15049D) or Proinflammatory Panel 2 (Meso Scale Discovery, K15053D) according to the manufacturer’s instructions.

### Draq7 death kinetics in tri-culture

2× treatments described in the previous section had Draq7 (Abcam, ab109202) added at 1:150. Cells were treated as described and imaged once per hour on the Incucyte S3. Images were analyzed using Incucyte software.

### Ferroptosis induction in HAP1 cell lines

*SEC24B* KO and isogenic control Hap1 cell lines (Horizon Discovery, HZGHC001222c002 and C631) were plated at 2.5 × 10^4^ cells per well of a 96-well plate in HAP1 media (Iscove’s Modified Dulbecco’s Medium, Gibco, 12440-053) + 20% FBS (Gibco, A38400-01). Twenty-four hours after plating, cells were treated with 1,600 µM FeSO_4_ + 1 µM RSL3 for 24 hours depending on the experiment. Cell death was tracked by Draq7 (Abcam, ab109202).

### Draq7 death kinetics for immortalized microglia and HAP1 cell lines

Draq7 (Abcam, ab109202) was added to treatments at 1:300. Cells were imaged on the Incucyte S3 once per hour. Images were analyzed using Incucyte software.

### Genome-wide CRISPR screen sgRNA lentiviral library production

Lenti-X 293T (Takara Bio, 632180) cells were thawed in complete Lenti-X 293T media (DMEM (Millipore, D5796) + 10% Tet-Free FBS (Takara Bio, 631101) + 1× penicillin–streptomycin (Millipore, TMS-AB2-C)) on an uncoated T75 flask. Once cells reached 80% confluency, cells were re-plated at 1 × 10^6^ cells per 10-cm dish in 8 ml of Lenti-X 293T media onto ten dishes. Cells were grown to 80–90% confluence. Each dish was transduced with one vial of Guide-it Genome-Wide sgRNA Library Transfection Mix (Takara Bio, 632650) as per the manufacturer’s instructions. Twenty-four hours after transduction, an additional 6 ml of media was added to each dish, for a total of 14 ml per dish. Forty-eight hours after transduction, supernatants were collected from each dish. Two dishes’ supernatants were pooled, creating five total libraries. Then, 8 ml of media was added back to each dish, and the supernatants were stored at 4 °C. Seventy-two hours after transduction, the remaining 8 ml of supernatant was collected from each dish and added to the respective stored supernatants, for a total of 44 ml per library. The libraries were centrifuged at 4 °C at 500 *g* for 10 minutes. Next, 200 µl was removed to determine viral titer, and the remaining was aliquoted and stored at −80 °C. Viral RNA was isolated with NucleoSpin RNA virus (Takara Bio, 740956) according to the manufacturer’s instructions, provided in Guide-it CRISPR Genome-Wide sgRNA Library System (Takara Bio, 632646). Viral titer was determined with Lenti-X qRT–PCR Titration Kit (Takara Bio, 631235) according to the manufacturer’s instructions.

### Genome-wide CRISPR screen

The human microglia cell line was plated in T150 flasks (BD Falcon, 355001) at 20 million per flask in 25 ml of complete microglia media and placed at 37 °C in a 5% CO_2_ incubator. Twenty-four hours after plating, puromycin (Takara Bio, 631305) was added at 5 µg ml^−1^. Cells were grown in 5 µg ml^−1^ of puromycin for 11 days. A full media exchange was performed without puromycin. Four days after removal of puromycin, Nunc non-treated T175 flasks (Thermo Fisher Scientific, 159926) were coated with 9 µg ml^−1^ of retronectin (Takara Bio, T110B) and incubated overnight at 4 °C. The next day, retronectin coating was removed and blocked in 2% BSA (Sigma-Aldrich, A7030) for 30 minutes at room temperature. Plates were washed 1× with PBS. Plates were coated with sgRNA pool at 60 multiplicity of infection (MOI) for 6 hours at 37 °C in a 5% CO_2_ incubator. After 6 hours, viral supernatants were removed, and cells were added at 1.67 × 10^7^ cells per flask across six flasks for a total of 1 × 10^8^ cells. Cells were placed at 37 °C in a 5% CO_2_ incubator. Twenty-four hours after transduction, cells were treated with 100 µg ml^−1^ of hygromycin B (Takara Bio, 631309). Cells were exposed to 100 µg ml^−1^ of hygromycin B for 8 days. Cells were lifted as previously described and plated to 20 Nunc EasYFlasks, 75 cm (Thermo Fisher Scientific, 156499), at 5 × 10^6^ cells per flask per replicate. Twenty-four hours after re-plating, cells were treated with 10× solutions (vehicle treatment or 1.6 mM FeSO_4_ + 10 µM RSL3) for either 8 hours or 24 hours. After treatment, cells were washed 2× with room temperature PBS, and media was replaced with complete microglia media. Two days after treatment, the vehicle control flasks had DNA from 1 × 10^8^ cells per replicate isolated with NucleoBond CB 500 Kit (Takara Bio, 740509) according to the manufacturer’s instructions. The 8-hour and 24-hour ferroptosis-treated cultures were allowed to re-grow for 4–10 days. Once sufficiently re-grown, DNA from 1 × 10^8^ cells were isolated with NucleoBond CB 500 Kit. DNA was stored at −80 °C.

### CRISPR screen analysis

The sgRNA sequences were amplified using Guide-it CRISPR Genome-Wide Library PCR Kit (Takara Bio, 632651) and subjected to the high-throughput amplicon sequencing on NextSeq 500. Then, 20 bp of sgRNA sequences were first extracted using Cutadapt. The sgRNA counting and hit generation were done in MAGeCK version 0.5.9, and the downstream analysis was performed by MAGeCKFlute version 1.10.0. The PCA plots were generated using edgeR and Glimma. Hits from the positive selection with *P* < 0.05 from each condition were imported into and intersected in RStudio. PANTHER was used for the GO enrichment analysis. Each condition has two biological replicates.

### Screen of commercial ferroptosis-related compounds

The human microglia cell line was plated at 1,000 cells per well of a 384-well plate. Twenty-four hours after plating, the cells were co-treated with the commercial ferroptosis compound library (Selleck Chemicals, L6400) at 10 µM or 2 µM, depending on the stock concentration of 10 mM or 2 mM, and 1,600 µM FeSO_4_ + 1 µM RSL3—10 µM for 535 of the compounds and 2 µM for 11 of the compounds. Twenty-four hours after treatment, cell survivability was determined by CellTiter-Glo (Promega, G9241). Compounds were tested in triplicate.

### Quantification and statistical analysis

All quantification and statistical analyses were completed in GraphPad Prism version 9.1.2, Harmony imaging version 4.9, FlowJo version 10.7.1, Cell Ranger version 4 and Zoom 2018A version 6.2.9200.0, as described in the figure legends and methods. In brief, tri-culture biological replicates were considered independently differentiated cultures, each with 1–3 technical replicates as individual wells. Statistical analysis across conditions was measured using one-way or two-way ANOVA. Dunnett, Tukey or Sidak post hoc tests were used as appropriate as indicated in the figure legends. For gene expression change in scRNA-seq or single-nucleus sequencing (nucSeq), log fold change cutoffs were >1.2 log-fold and >.1 log_2_-fold, respectively, with adjusted *P* < 0.05, as described in the figure legends and methods. For the genome-wide CRISPR screen, two independent viral pools were used across two replicate cell cultures for each viral pool. For all experiments involving the *SEC24B* KO HAP1 line and its isogenic control, each biological replicate was considered as an independent culture. Unpaired two-tailed *t*-test and one-way ANOVA with a Dunnett post hoc was used as indicated in the figure legends. The compound screen was performed with three technical replicates. Data for tri-culture death kinetics, FTH expression, cell counts and neuronal lipid peroxidation were log-transformed to account for baseline shifts. For all panels where statistical significance is indicated, *P < 0.05, **P < 0.01, **P < 0.001 and ****P < 0.0001. Bar graphs display individual data points and report the data as mean ± s.e.m. No statistical methods were used to pre-determine sample sizes, but our sample sizes are similar to those reported in previous publications^39,70^. Data distribution was assumed to be normal, but this was not formally tested. Wells were randomly chosen on each plate for each condition. Data collection and analysis were not performed blinded to the conditions of the experiments. Blinding was not relevant to our study as all measurements were automated such that bias would not be introduced. No data were excluded from the analysis.

### Reporting Summary

Further information on research design is available in the Nature Portfolio Reporting Summary linked to this article.

## Online content

Any methods, additional references, Nature Portfolio reporting summaries, source data, extended data, supplementary information, acknowledgements, peer review information; details of author contributions and competing interests; and statements of data and code availability are available at 10.1038/s41593-022-01221-3.

### Supplementary information

Supplementary Information Supplementary Materials and Methods, Tables 1 and 2, Figs. 1 and 2 and References.

Reporting Summary

Supplementary Video 1 Ferroptosis in tri-culture with microglia.

Supplementary Video 2 Ferroptosis in tri-culture without microglia.

## Data Availability

All data associated with this study are present in the paper or Supplementary Materials. AMP PD data are available through the Terra platform (https://amp-pd.org/tools) by request and require approval for access through the AMP PD data use agreement. Target ALS datasets are available through https://www.targetals.org/resource/genomic-datasets/. The Access and Compliance Team membership is made up of representatives from the National Institutes of Health and includes representatives from the National Institute of Neurological Disorders and Stroke (NINDS) and the National Institute on Aging (NIA). NINDS representatives are responsible for all access and compliance activities related to the AMP PD unified cohorts, and NIA representatives are responsible for all access and compliance activities related to the Global Parkinson’s Genetics Program (GP2) cohorts. GRCh38 human reference genome is available at https://www.ncbi.nlm.nih.gov/assembly/GCF_000001405.40. Transcriptomic data are available as cited or accessible at https://www.synapse.org/ (Synapse ID syn41699699 for the iPSC tri-culture processed gene counts matrixes). FASTQ files are also available upon request at syn40800403.

## Code availability

No custom analysis code was used in this study.
