# Supplementary material for: Microglia ferroptosis is regulated by SEC24B and contributes to neurodegeneration
Source: Nat Neurosci. 2022 Dec 19;26(1):12–26. doi: 10.1038/s41593-022-01221-3 (PMC9829540; doi:10.1038/s41593-022-01221-3)
Supplement: Supplementary file 1 — Supplementary Materials and Methods, Tables 1 and 2, Figs. 1 and 2 and References. [file 41593_2022_1221_MOESM1_ESM.pdf]

nature neuroscience

Article

https://doi.org/10.1038/s41593-022-01221-3

Microglia ferroptosis is regulated by SEC24B and contributes to neurodegeneration

In the format provided by the authors and unedited

Supplementary Materials

Supplementary materials and methods

FACS on tri-culture

Cells were washed 2x with PBS. Cells were then treated with 0.25% Trypsin + EDTA (Sigma T4049 for 6min in 37°C 5% CO_2 incubator. 1 volume of PBS + 2% FBS (Gibco A38400-01) was added to quench. Cells were combined from two wells and put through 40µm cell strainers (BD Falcon 352235). Strained cell suspension was placed in a 1.7ml eppendorf tube on ice. Cell suspensions were spun at 4°C for 5min at 528xg. Supernatant was removed and cells were resuspended. Collected cells were resuspended in flow cytometry staining buffer (BD 554656) containing anti-GLAST (Miltenyi 130-118-344, 1:50) PE-conjugated antibody for 10 minutes at room temperature. Samples were washed once, resuspended, and analyzed on a BD LSRII instrument. Results were analyzed in FlowJo software (Treestar).

Immunocytochemistry

Post treatment, supernatant was collected, and cells were washed 2x with RT PBS. Cells were fixed with 4% PFA (Fisher Scientific 50980495) for 15min at RT. Cells were washed 3x with PBS before being stored in PBS at 4°C. Cells were blocked with 5% BSA (Sigma A7030) + 0.1% Triton-X 100 (Sigma T9284) in PBS for 1hr at RT. Primary and secondary antibodies were prepared in blocking buffer. Cells were incubated in primary antibodies overnight at 4°C. Cells were washed 4x for 5min with PBS + 0.1% Tween20 (Sigma P9416). Cells were incubated in secondary antibodies for 1hr at RT in the dark. DAPI (Life Technologies D3571) was added during secondary incubation. Cells were washed 3x for 5min with PBS + 0.1% Tween20 and 1x in PBS. Cells were imaged and analyzed on Opera Phoenix. Antibodies used for immunocytochemistry: mouse anti-ferritin (RnD Systems MAB93541, 1:500); rabbit anti-IBA1 (Wako 019-19741, 1:500); chicken anti-MAP2 (abcam ab92434, 1:1000); chicken anti-GFAP (Millipore AB5541, 1:500); mouse anti-βIII tubulin (Millipore MAB1637); donkey anti-mouse (invitrogen A21202, 1:500); donkey anti-rabbit (invitrogen A31572, 1:500); goat anti-chicken (invitrogen A21449, 1:500).

Click-it lipid peroxidation

Click-it lipid peroxidation imaging kit (Invitrogen C10446) was used per manufacturer's instructions. 50mM Linoleamide alkyne (LAA) was added 1:500 to previously described 2x treatments for a 100mM 2x stock. Immunocytochemistry was performed as previously described after Click-it LAA detection was performed

Human immortalized microglia cell culture

Human immortalized microglia (abm T3451) were cultured in complete microglia media: (DMEM Millipore D6546) +10% Tet-free FBS (Takara bio 631101) + 1x L-glutamine (Millipore #TMS-002-C) + 1x pen/strep (Millipore #TMS-AB2-C).

Ferroptosis induction in human immortalized microglia

The human microglia cell line (abm T3451) was plated at $5x10^3$ cells/ well of a 96 well plate. 24hrs post plating, cells were treated. Treatments included: 400µM $FeSO_4$, 800µM $FeSO_4$, and 1600µM $FeSO_4$, 1µM RSL3, µM benzothiazine, and 100nM lip-1. Cells were treated for 2,4, or 24 hours depending on the experiment. Cell viability was analyzed by Cell titer glo (Promega G9241).

Lipidomics cell preparation

The human microglia cell line was plated at $5x10^6$ cells / T75 plate (Thermo Fisher Scientific 156499) in complete microglia media: (DMEM Millipore D6546) +10% Tet-free FBS (Takara bio 631101) + 1x L-glutamine (Millipore #TMS-002-C) + 1x pen/strep (Millipore #TMS-AB2-C). 24 hours later, cells were treated with 10x solutions. 10x solutions were: 1:500 DMSO, 1.6mM $FeSO_4$ + 1:500 DMSO, 1.6mM $FeSO_4$ + 10µM RSL3, 1.6mM $FeSO_4$ + 10µM RSL3 + 1µM lip-1, 1.6mM $FeSO_4$ + 10µM RSL3 + 10µM benzothiazine, 10µM RSL3, and 1µM lip-1. 2hrs and 4hrs post treatment, cells were collected. Cells were washed 2x with RT PBS. Cells were then treated with 0.25% Trypsin + EDTA for 5 min in 37°C incubator. Cells were quenched with 1 volume of complete microglia media. Cells were spun down at 4°C at 338xg for 5 min. Cells were washed and re-spun 2x with ice cold PBS. $1x10^6$ cells per sample were pelleted, supernatant was aspirated, flash frozen, and stored at -80°C before being sent for lipidomics analysis.

Lipidomic extraction solution preparation: 50 mL methanol was mixed with 50 mL chloroform. 0.2 mL acetic acid was added. 12(S)-HETE-d8 internal standard was added to get a final concentration of 10ng/mL. 15:0-18:1-d7-PE was added to get a final concentration of 20ng/mL.

Unhydrolyzed sample preparation for lipidomics

Cell pellet of $1x10^6$ cells was resuspended with 0.2 mL ice cold water and resuspended by pipetting gently. The sample was transferred to a glass tube (with cap. 11 mL, 16 mm x 100 mm). 2 mL of extraction solution and 0.8 mL of water was added. Samples were vortexed (VWR, VX-2500 Multi-tube vortexer) for 5 min at the speed of 10 (max speed). Sample were incubated in the cold room for 20 min. Tubes were vortexed and then centrifuged for 10 minutes at 3000 rcf on Eppendorf 5810R centrifuge. The bottom layer was transferred to a new glass tube by using glass pasteur pipette and the organic solvent was dried by nitrogen. The dried lipid was resuspended in 100 µL methanol. Samples were transferred to LC-MS sample vials.

Hydrolyzed sample preparation for lipidomics

Cell pellet of $1x10^6$ cells was resuspended with 0.2 mL ice cold water and resuspended by pipetting gently. The sample was transferred to a glass tube (with cap. 11 mL, 16 mm x 100 mm). 2 mL of extraction solution and 0.8 mL of water was added. Samples were vortexed (VWR, VX-2500 Multi-tube vortexer) for 5 min at the speed of 10 (max speed). Sample were incubated in the cold room for 20 min. Tubes were vortexed and then centrifuged for 10 minutes at 3000 rcf on Eppendorf 5810R centrifuge. The bottom layer was transferred to a new glass tube by using glass pasteur pipette. 50 ul 10 M KOH solution was added, mixed, and incubated at 37 °C for 2 h. 0.6 ml water was added, vortexed 2-5 min, and incubated in cold room for 20 min. Tubes were vortexed and then centrifuged for 10 min. The bottom layer was transferred to a new glass tube by

using a glass pasteur pipette. The organic solvent was dried by nitrogen. The dried lipid was resuspended in 100 µL methanol. The sample was transferred to an LC-MS sample vial.

H(p)ETE analysis by LC-MS

LC separation was performed with an ACQUITY UPLC system. The multiple-reaction-monitoring (MRM) spectra were obtained with a Sciex Triple Quad 6500 mass spectrometer. A Waters Premier Acquity BEH C18 column (2.1 mm × 150 mm, 1.7 µm) was used for the LC separation. The mobile phase consisted of (A) water/acetonitrile 75/25 with 10 mM ammonium acetate and (B) isopropanol/acetonitrile 50/50. The flow rate was 0.4 mL min–1. The solvent-gradient elution was changed as follows: 0 min, 0% B; 0.5 min, 0% B; 6 min, 50% B; 6.1 min, 100% B; 8 min, 100% B; 8.1 min, 0% B; 10 min, 0% B. Five microliter was injected. All data were acquired in negative-ion mode.

H(p)ETE-PE analysis by LC-MS

LC separation was performed with an ACQUITY UPLC system. The selected-ion-monitoring (SIM) spectra were obtained with Q-exactive HF high resolution mass spectrometer. Waters Acquity BEH HILIC column (1.7 µm, 2.1 x 150 mm) was used for the LC separation. The mobile phase consisted of (A) 96% ACN, 2% MeOH, 1% Acetic acid, 1% H2O, 5 mM Ammonium acetate and (B) 98% MeOH, 1% Acetic acid, 1% H2O, 5 mM Ammonium acetate. The flow rate was 0.3 mL/min. The solvent-gradient elution was changed as follows: 0 min, 5% B; 2 min, 5% B; 15 min, 50% B; 16 min, 50% B; 16.1 min, 50% B; 25 min, 5% B. Five microliters were injected into the UPLC system. All data was acquired in negative-ion mode.

Standard curves were made with HETE-PE(C38:4) and HpETE-PE (C38:4) following the same extraction/sample prep.

PE Profiling by High Resolution MS

LC conditions were same as H(p)ETE-PE analysis. The profiling data (700-950Da in negative ion mode) were collected on Q-exactive HF high resolution mass spectrometer. PE class are eluted at a characteristic retention time on HILIC column (Waters Acquity BEH HILIC column 1.7 µm, 2.1 x 150 mm) and PE identity are assigned by retention time and exact mass (<5ppm).

Profiling data is processed (aligned, peak picked, and clustered) by Expressionist 14.0 (Genedata, MS refiner). And statistical analysis (P-value and Fold change) is performed in Analyst (Expressionist 14.0, Genedata).

PD blood bulk RNAseq analysis

Data Availability: Unified AMP-PD Cohorts data is restricted and requires authorization for access: https://amp-pd.org/. Information and access to the Terra computing platform can be found at https://terra.bio/. Terra is developed by the Broad Institute of MIT and Harvard in collaboration with Verily Life Sciences. The Terra system was used for access to AMP-PD data and for computing. AMP-PD is a public-private partnership between the United States National Institutes of Health, non-profit organizations, and pharmaceutical and life sciences companies which aims to identify and validate new, impactful biological targets for PD therapeutics. For this analysis, PD

Case and Control subjects enrolled in two large cohorts of the Accelerating Medicines Partnership - Parkinson's Disease (AMP-PD) Consortia were included: Parkinson’s Disease Biomarker Program (PDBP; 780 PD Cases, 504 Controls) and the Parkinson’s Progression Markers Initiative (PPMI; 816 PD cases, 617 Controls) (Fig. 5A). Although both PPMI and PDBP are case-control cohorts whose data are included in AMP-PD, it should be noted that these cohorts differ with respect to their inclusion criteria: PPMI inclusion criteria restricts enrollment to only those PD patients who have been diagnosed 2 years or less, whose baseline Hoehn & Yahr stage is I or II, and who are not expected to require medication for at least 6 months [1]. In contrast, PDBP enrolls PD patients across Hoehn & Yahr Stages, thus baseline patient data from PDBP includes a range of progression stages [2].

Data management, preprocessing and differential expression analysis were performed in Google Terra workspaces using R 3.6 and Python 3.7. Baseline differential gene expression analyses were performed for peripheral blood RNA sequencing data obtained at the point of study enrollment (baseline; BL) using the DESeq2 package [3]. The pathway analysis for DE gene sets was performed using Ingenuity Pathway Analysis software using adjusted p-value cutoff for pathway enriched <0.05 [4]. Transcriptomic quality control for samples was performed by the AMP-PD consortia. The decision tree for transcriptomics quality control is available on www.amp-pd.org. In this analysis, we have used the raw counts' output of Salmon v0.11.3 (PMCID: PMC5600148) for the abundant transcript estimation. Samples that were identified to have less than 50M reads, and outliers from the basic principal components analysis were flagged by the AMP-PD consortia and were not included in our further analysis. The DESeq2 R package (version 1.26.0) was used to identify differentially expressed genes among PD cases versus controls. DESeq2 is a method for differential expression analysis which may be applied to RNA-seq read counts per gene to estimate fold changes across conditions, in this case differences in gene expression measured from peripheral blood samples obtained from PD case and normal control subjects at point of study enrollment. As this is an exploratory analysis and validation, we have performed the analysis for the males and females study cohorts separately to identify potential differences between them. The design formula used for each differential expression analysis included age group as a covariate, sequencing plate number as batch variable, and diagnosis for the case vs. control comparison. Pre-processing and normalization of all RNAseq samples were done separately for each analysis for all expressed genes. Sample normalization and statistical testing for against a null hypothesis of no difference in gene expression between PD case and control subjects was performed using the Wald test, with p-values adjusted for multiple testing using the Benjamin and Hochberg method. Adjusted p-values of 0.05 and normalized gene log fold change in expression between conditions were then used as cutoffs for identification of protein-coding genes for use in downstream pathway analyses.

Single nucleus RNASeq microglia analysis

Control (n=3; 21,531 nuclei) and PD (n=3; 31,069 nuclei) snRNA samples data, sequencing, and analyses been previously reported [5, 6]. For initial broad types clustering, count matrices with nucleus barcodes and gene labels were loaded with Python version 3.5 and R version 4.0/RStudio for sample integration and unsupervised clustering using Seurat Package version 4.0. For Quality

Control (QC), nuclei were first filtered using the Scrublets [7] package for doublet detection then filtered following standard protocols based on examination of violin plots. Cutoffs 200 < nFeature_RNA and percent.mt < 5 were used. Filtered matrices were then individually log-normalized by sample according to standard Seurat workflows. Sample integration was performed in Seurat using the RunHarmony function with 2000 variable features [8] and clustering resolution 0.8. Cluster-level markers were identified using the FindMarkers function and marker enrichment as well as cluster-level expression of major cell type markers were used to annotate cells contained within each cluster and confirm broad type assignments with selected markers shown on violin plots in the supplemental materials (Suppl. Fig. 4).

Microglia Coexpression Network Profiling: Nuclei identified as microglia by canonical marker expression were then subsetted from the Seurat object by cluster name (“Microglia”) (Control, n = 659 nuclei; PD, n = 1652 nuclei).

Weighted Gene Co-expression Network Analysis for single cell (hdWGCNA): hdWGCNA was performed in R using the hdWGCNA package [9] and development package version in R hdWGCNA 0.1.1.9005 (2022-06-17). The standard hdWGCNA was applied to the gene-gene co-expression matrix generated from the normalized cell-gene arrays from the integrated Seurat object containing microglia nuclei from the triculture experiments for the vehicle and iron + RSL3 conditions. A soft thresholding power = 3 was selected based on initial analyses. For each gene co-expression module identified by hdWGCNA, we then calculated the correlation between the module eigenvector and the dummy-encoded disease trait (vehicle or iron + RSL3) in order to assess if any of these gene coexpression modules might be significantly correlated with disease state. Module genes co-expressed more actively in nuclei from a significantly correlated trait group are those which are positively correlated; negative module correlation suggests less active co-expression by trait. We then subsetted microglia from the putamen snRNAseq analysis and applied the hdWGCNA package function ProjectModules to transfer module gene sets to putamen data. Dot Plots of module scores were then used to examine differences in module gene expression patterns between controls and PD patients as for the vehicle and iron + RSL3 treated microglia.

PD tissue cytokine analysis

MSD assay (V-PLEX Proinflammatory Panel 1 Human Kit- K15049D) was used to quantify protein expression of IL-8, IL-6, TNFa and IL-1b in homogenized brain samples from control and PD samples. Substantia nigra (n= 11 con/ 9 PD patients), BA24 (n= 8 con/ 8 PD patients) and BA25 (n= 8 con/ 7 PD patients) (obtained from Harvard Brain Tissue Resource Center) were homogenized in 1x RIPA buffer (Boston BioProducts BP-115) with cOmplete protease inhibitor (Roche 11697498001) on ice and briefly vortexed and sonicated. Samples were diluted 1:1 and the MSD protocol was followed.

TargetALS

Raw fastq RNA-Seq data files (1072 total) were provided by the NYGC ALS Consortium (Target ALS Release, June 2020) (https://www.targetals.org/research/resources-for-scientists/resource-genomic-data-sets/). These fastq files were generated using human postmortem tissue samples

from the Target ALS postmortem tissue core and processed for RNA-Seq as previously described [10]. The raw fastq files were processed by Qiagen in OmicSoft ArrayStudio RNA-Seq analysis pipeline (version 10.1.1.3). In brief, the fastq files had quality control performed and then aligned to the Genome Reference Consortium Human Build 37 (GRCh37, GPL16791) using the proprietary OmicSoft Aligner [11]. After alignment, the gene level RPKM/FPKM/counts were determined using the EM algorithm in ArrayStudio as described previously [12]. Finally, pairwise differentially expressed genes were calculated using the DESeq2 v1.10.1 [3] in ArrayStudio comparing tissue-specific ALS patients vs. non-neurological controls. ALS patients with multiple neurological conditions were excluded from pairwise analysis. Heatmaps were generated using Excel. Differentially expressed genes (DEGs) were considered significant with a padj < 0.05.

Protein Isolation

SEC24B KO and isogenic control Hap1 cell lines (Horizon Discovery HZGHC001222c002 & C631) were plated on uncoated 6-well plates (Corning 3516) at $2x10^6$ cells per well. 24hrs post-plating, cells were washed 2x with RT PBS. Cells were then treated with 0.25% Trypsin + EDTA for 5 min in 37°C incubator. Cells were quenched with 1 volume of HAP1 media (Iscove’s Modified Dulbecco’s Medium (IMDM) (Gibco 12440-053) + 20% FBS (Gibco A38400-01)). Cells were spun down at RT at 338xgfor 5 min. Supernatant was aspirated and cells were resuspended in PBS and spun down at RT at 338xg for 5 min. Supernatant was aspirated and cell pellet was resuspended in 100uL 1x RIPA buffer (Boston BioProducts BP-115) with cOmplete protease inhibitor (Roche 11697498001). Cells were briefly vortexed and left on ice for 10 min. Lysates were sonicated for 10sec with VirSonic 100 wand sonicator. Lysates were then spun at 14,000xg for 10min at 4ºC. Supernatants were collected and stored at -80ºC. Protein concentration was determined by BCA (Thermo Scientific 23227).

Western blot

Protein samples were prepared in Laemmli SDS sample buffer (Boston BioProducts BP-111R)). 20ug samples were boiled for 10min at 95°C and then run in 4-12% Bis-Tris gels (Invitrogen NP0321BOX) in 1x MOPS running buffer (Life technologies NP0001) at 150V for 1hr. Gel was dry transferred with iBlot2 (Invitrogen). Blots were cut at specific kD sizes to allow blotting for multiple proteins from the same gel. Blots were blocked for 1hr at RT in Intercept Blocking Buffer (Li-Cor 927-60001). Blots were incubated in primary antibody solution (Intercept Blocking Buffer) with rabbit anti-SEC24B (Cell Signaling Technology 12042S, 1:500), rabbit anti-LC3B (Sigma L7543, 1:1,000), guinea pig anti-p62 (MBL PM066, 1:1,000) or mouse anti-β-actin (Sigma A5441, 1:5,000) at 4°C overnight. Blots were washed 4x for 5 min with TBS-T (0.2% Tween-20). Blots were incubated in Secondary antibody solution (Intercept Blocking Buffer + 0.2% Tween-20) with Donkey anti-Mouse 680 (Li-Cor 926-68072, 1:2,000), Donkey anti-Rabbit 800 (Li-Cor 926-32213, 1:2,000), or Donkey anti-guinea pig 800 (Li-Cor, 926-32411, 1:2,000) in the dark at RT for 1hr. Blots were washed 3x for 5min with TBS-T and 1x with TBS for 5min. Blots were imaged on Odyssey CLx. Images were analyzed on ImageJ.

RNA isolation and qRT-PCR

RNA was isolated with RNeasy Plus Mini Kit (Qiagen 74134) according to manufacturer's instructions. cDNA was synthesized from RNA with SuperScript VILO (Invitrogen 11755-050). RNA expression was quantified with taqman probes (Thermo Fisher Scientific) for *SEC24B* (Hs00197035_m1) and *RPL37A* (Hs01102345_m1) and TaqMan Gene Expression Master Mix (Applied Biosystems 4369016). 30ng of cDNA was used per well, with three technical replicates per sample. qRT-PCR was run on Applied Biosystems QuantStudio 7 Flex. Expression levels were normalized to *RPL37A*.

BODIPY FACS

SEC24B KO and isogenic control Hap1 cell lines (Horizon Discovery HZGHC001222c002 & C631) were plated on uncoated, TC-treated T25 flasks (BD falcon 353109) at $1x10^6$ cells per flask in 4.5mL of HAP1 media (Iscove's Modified Dulbecco's Medium (IMDM) (Gibco 12440-053) + 20% FBS (Gibco A38400-01)). 24hrs post-plating, cells were treated with 500uL 10x 1.6mM FeSO4 + 10µM RSL3 or DMSO (1:1000) vehicle control. 1 or 3hrs post-treatment, 5uL of 10mM Image-iT (BODIPY 581/591 C11) (Invitrogen C10445) was added to the cultures. 1hr post Image-iT addition, the cells were collected, for either a 2hr or 4hr treatment time. Cells were washed 1x with PBS and then treated with 0.25% Trypsin + EDTA for 5 min in 37°C incubator. Cells were quenched with 1 volume of HAP1 media. Cells were collected and spun down at RT at 338xg for 5 min. Supernatants were aspirated and cells were resuspended in FACS buffer (PBS + 1.5% BSA (Sigma A1470) + 5mM EDTA (Nalgene AM9260G) + 5% FBS (Sigma F2442)). Cell suspensions were placed through a 40µm cell strainer on a 5mL round-bottom tube (BD falcon 352235). Cells were processed on Sony SH800S cell sorter under the FITC and PE filters. Live, single cells were selected for analysis using forward and side scatter. The percent of $FITC^{bright}$ PE+ cells was analyzed for each sample, with gates determined using unstimulated controls.

Autophagy induction

SEC24B KO and isogenic control Hap1 cell lines (Horizon Discovery HZGHC001222c002 & C631) were plated on uncoated, TC-treated 10cm dishes (Corning 430167) at $8x10^6$ cells per plate in 10mL of HAP1 media. 24hr post -plating cells were serum-starved (HAP1 media without FBS) and treated with 100µM chloroquine (R&D systems 4109/50) for 6hrs. Cells were washed 2x with PBS. 250uL 1x RIPA buffer (Boston BioProducts BP-115) with cOmplete protease inhibitor (Roche 11697498001) was added directly to each plate. Cells were collected, vortexed briefly, and placed on ice for 10min. Lysates were sonicated for 10sec with VirSonic 100 wand sonicator. Lysates were then spun at 14,000xg for 10min at 4ºC. Supernatants were collected and stored at -80ºC.

Erastin induction for death kinetics

SEC24B KO and isogenic control Hap1 cell lines (Horizon Discovery HZGHC001222c002 & C631) were plated on uncoated, TC-treated 96-well plates (Corning 3595) at $4.7x10^4$ cells/cm^2 in 100µL of HAP1 media. 24hr post -plating cells were treated with vehicle (DMSO) or 10µM erastin and Draq7 (abcam ab109202). Cells were imaged in the incucyte S3 once an hour. Images were analyzed on incucyte software.

Erastin induction for protein isolation

SEC24B KO and isogenic control Hap1 cell lines (Horizon Discovery HZGHC001222c002 & C631) were plated on uncoated, TC-treated 6-well plates (Corning 3516) at $4.7x10^4$ cells/cm^2 in 2mL of HAP1 media. 24hr post -plating cells were treated with vehicle (DMSO) or 10µM erastin for 24hrs. Cells were washed 1x RT PBS. 150uL 1x RIPA buffer (Boston BioProducts BP-115) with cOmplete protease inhibitor (Roche 11697498001) was added directly to each plate. Cells were collected, vortexed briefly, and placed on ice for 10min. Lysates were then spun at 14,000xg for 10min at 4ºC. Supernatants were collected and stored at -80ºC.

Ferritin ELISA

Lysates from autophagy induction and Erastin induction were run on Ferritin human ELISA kit (Invitrogen EHFTL) according to manufacturer's instructions.

Free iron assay

Lysates from 24hr Erastin induction were run on Iron assay kit (Sigma MAK025) according to manufacturer's instructions. Only free, reduced iron was measured.

Supplementary Tables

Table S1. Target ALS sample numbers

Table S1. Target ALS Tissue Breakdown			
**Tissues**	**ALS**	**Non-Neurological Control**	**Both**
cerebellum	114	13	127
frontal cortex	95	13	108
motor cortex lateral	80	11	91
motor cortex medial	80	12	92
motor cortex unspecified	9	1	10
motor cortex	169	24	193
occipital cortex	46	6	52
cervical spinal cord	92	12	104
lumbar spinal cord	79	10	89
thoracic spinal cord	44	8	52
spinal cord	215	30	245

Table S2. Full hit list from ferroptosis compound screen

compound name	percent recove	conc used	target	pathway
Xanthotoxol	158.3764471	10uM DMSO	Inflammation/Immunology	5-HT Receptor
BAY 87-2243	87.10295311	10uM DMSO	HIF	Angiogenesis
Sesamol	97.73483611	10uM DMSO	Others	antioxidant
Ethoxyquin	75.584162	10uM DMSO	Others	antioxidant
Diphenylamine Hydrochloride	82.05480531	10uM water	Others	antioxidant
Lauryl gallate	70.36480757	10uM DMSO	Others	antioxidant
(+)-Delta-Tocopherol	77.59477972	10uM DMSO	Others	antioxidant
Octyl gallate	74.85572818	10uM DMSO	Others	antioxidant
Cyclic Pifithrin-α hydrobromide	72.77337001	10uM DMSO	p53	Apoptosis
WNK463	70.42145001	10uM DMSO	Serine/threonin kinase	Apoptosis
Tenovin-1	149.1821169	10uM DMSO	E3 Ligase ,p53	Apoptosis
Necrostatin-1	83.85006199	10uM DMSO	TNF-alpha	Apoptosis
3,6'-Disinapoyl sucrose	76.23339408	10uM DMSO	3,6'-Disinapoyl sucrose	Bcl-2
Isosilybin	73.41418741	10uM DMSO	CYP3A474μM	Cancer
Schisandrin C	95.48330333	10uM DMSO	Cancer	Cancer
Epiberberine	89.4575386	10uM DMSO	GMNN - geminin, DNA replication inhibitor (human)	DNA replication
Equol	72.21767315	10uM DMSO	Estrogen/progestogen Receptor	Endocrinology & Hormones
Psoralidin	82.98420834	10uM DMSO	Estrogen/progestogen Receptor	Endocrinology & Hormones
Curcumin	129.6421671	10uM DMSO	NF-κB,HDAC,Histone Acetyltransferase,Nrf2	Epigenetics
Boldine	87.34264647	10uM DMSO	FXR receptor	FXR receptor
Schisanhenol	82.17574014	10uM DMSO	Others	glucuronosyltransferases
Olivetol	71.0489686	10uM DMSO	Cannabinoid Receptor	GPCR & G Protein
Thymol	77.85836852	10uM DMSO	Immunology & Inflammation related	Immunology & Inflammation
2-Acetylphenothiazine (ML171)	70.71460427	10uM DMSO	NADPH-oxidase	Immunology & Inflammation
Trolox	93.4535961	10uM DMSO	Vitamin	Metabolism
Galangin	83.13078825	10uM DMSO	P450 (e.g. CYP17)	Metabolism
ML355	75.13140889	10uM DMSO	Lipoxygenase	Metabolism
Ferrostatin-1 (Fer-1)	80.03178369	10uM DMSO	Ferroptosis	Metabolism
GKT137831	79.43502429	10uM DMSO	NADPH-oxidase	NADPH-Oxidase
Levocetirizine Dihydrochloride	84.72345445	10uM DMSO	Histamine Receptor	Neuronal Signaling
Jatrorrhizine	78.91407161	10uM DMSO	AChR	Neuronal Signaling
8-OH-DPAT (8-Hydroxy-DPAT)	83.49121491	10uM DMSO	5-HT Receptor	Neuronal Signaling
JSH-23	96.08833966	10uM DMSO	NF-κB	NF-κB
ATP	111.0616153	10uM DMSO	Others	Others
Rhapontigenin	191.1995477	10uM DMSO	Others	proliferation
Ferulaldehyde	75.0495201	10uM DMSO	Others	proliferation
Crizotinib (PF-02341066)	88.45740205	10uM DMSO	ALK,c-Met	Protein Tyrosine Kinase
Zonisamide	78.20997873	10uM DMSO	Sodium Channel	Transmembrane Transporters
Demethoxycurcumin	97.50980203	10uM DMSO	iNOS	Wnt/β-catenin

Supplementary Figure

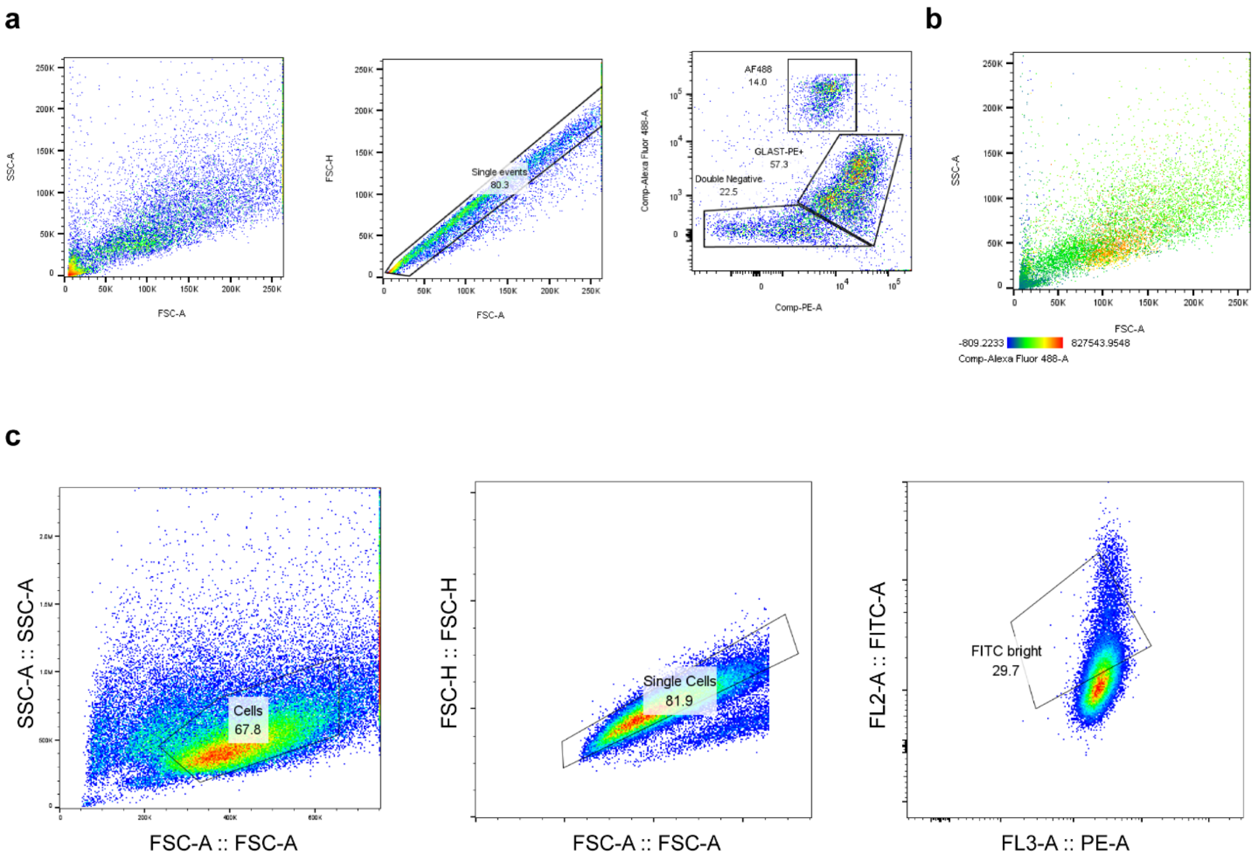

Supplementary Fig. 1. Gating strategies

a and **b**, gating strategies for live, single cells (**a**) and for alexa fluor 488 compensation (**b**) for flow cytometry analysis of cell composition in the tri-culture (Ext. Data Fig. 1a). **c**, gating strategy for live, single cells and FITC bright cells for HAP1 lipid peroxidation experiments (Fig. 6d and Extended Data Fig. 8e).

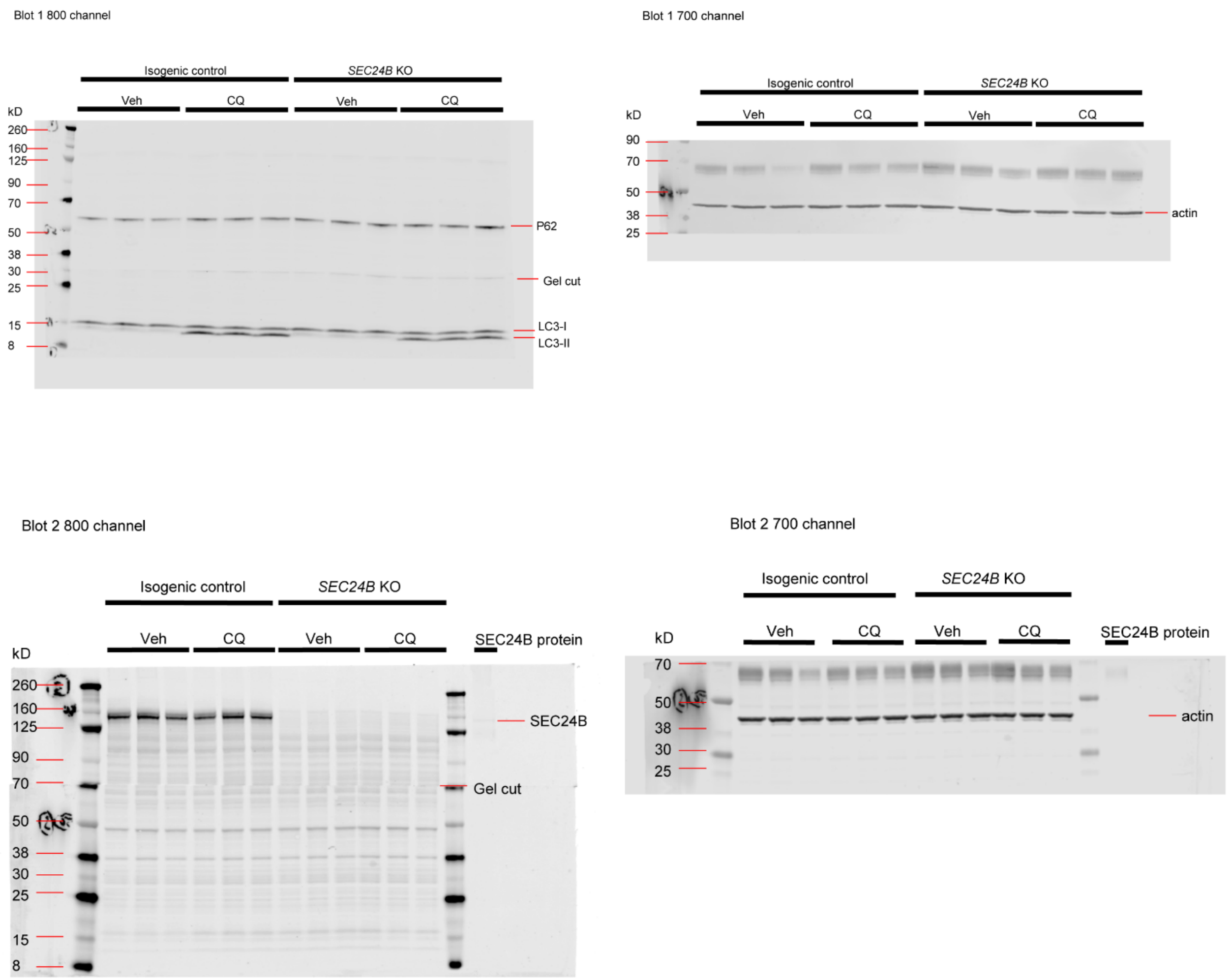

Supplementary Fig. 2. Full western blots

Blot 1 is for autophagy-related experiments, blotting for P62, LC3, and actin. Only one section of the blot was stained for in the 700 channel for actin. Blot 2 is for SEC24B staining. Only one section of the blot was stained for in the 700 channel for actin.

Supplementary References

1. Marek, K., et al., *The Parkinson Progression Marker Initiative (PPMI).* Progress in Neurobiology, 2011. **95**(4): p. 629-635.
2. Ofori, E., et al., *Parkinson's disease biomarkers program brain imaging repository.* NeuroImage, 2016. **124**(Pt B): p. 1120-1124.
3. Love, M.I., W. Huber, and S. Anders, *Moderated estimation of fold change and dispersion for RNA-seq data with DESeq2.* Genome Biology, 2014. **15**(12): p. 550.
4. Krämer, A., et al., *Causal analysis approaches in Ingenuity Pathway Analysis.* Bioinformatics, 2014. **30**(4): p. 523-530.

5. Teeple, E., et al., *Single Nuclei Sequencing of Human Putamen Oligodendrocytes Reveals Altered Heterogeneity and Disease-Associated Changes in Parkinson's Disease and Multiple System Atrophy.* bioRxiv, 2021: p. 2021.05.06.442967.
6. Pande, R., et al., *Single Cell Atlas of Human Putamen Reveals Disease Specific Changes in Synucleinopathies: Parkinson's Disease and Multiple System Atrophy.* bioRxiv, 2021: p. 2021.05.06.442950.
7. Wolock, S.L., R. Lopez, and A.M. Klein, *Scrublet: Computational Identification of Cell Doublets in Single-Cell Transcriptomic Data.* Cell Systems, 2019. **8**(4): p. 281-291.e9.
8. Korsunsky, I., et al., *Fast, sensitive and accurate integration of single-cell data with Harmony.* Nature Methods, 2019. **16**(12): p. 1289-1296.
9. Morabito, S., et al., *Single-nucleus chromatin accessibility and transcriptomic characterization of Alzheimer's disease.* Nature Genetics, 2021. **53**(8): p. 1143-1155.
10. Tam, O.H., et al., *Postmortem Cortex Samples Identify Distinct Molecular Subtypes of ALS: Retrotransposon Activation, Oxidative Stress, and Activated Glia.* Cell Reports, 2019. **29**(5): p. 1164-1177.e5.
11. Hu, X., et al., *pIRS: Profile-based Illumina pair-end reads simulator.* Bioinformatics, 2012. **28**(11): p. 1533-1535.
12. Li, Y. and J.C. Patra, *Genome-wide inferring gene–phenotype relationship by walking on the heterogeneous network.* Bioinformatics, 2010. **26**(9): p. 1219-1224.
