# Supplementary material for: Microglia ferroptosis is regulated by SEC24B and contributes to neurodegeneration
Source: Nat Neurosci. 2022 Dec 19;26(1):12–26. doi: 10.1038/s41593-022-01221-3 (PMC9829540; doi:10.1038/s41593-022-01221-3)
Supplement: Supplementary file 2 — Reporting Summary [file 41593_2022_1221_MOESM2_ESM.pdf]

nature portfolio

Corresponding author(s): Timothy R. Hammond

Last updated by author(s): October 12, 2022

Reporting Summary

Nature Portfolio wishes to improve the reproducibility of the work that we publish. This form provides structure for consistency and transparency in reporting. For further information on Nature Portfolio policies, see our Editorial Policies and the Editorial Policy Checklist.

Statistics

For all statistical analyses, confirm that the following items are present in the figure legend, table legend, main text, or Methods section.

n/a	Confirmed	
☐	☒	The exact sample size (*n*) for each experimental group/condition, given as a discrete number and unit of measurement
☐	☒	A statement on whether measurements were taken from distinct samples or whether the same sample was measured repeatedly
☐	☒	The statistical test(s) used AND whether they are one- or two-sided<br>*Only common tests should be described solely by name; describe more complex techniques in the Methods section.*
☐	☒	A description of all covariates tested
☐	☒	A description of any assumptions or corrections, such as tests of normality and adjustment for multiple comparisons
☐	☒	A full description of the statistical parameters including central tendency (e.g. means) or other basic estimates (e.g. regression coefficient) AND variation (e.g. standard deviation) or associated estimates of uncertainty (e.g. confidence intervals)
☐	☒	For null hypothesis testing, the test statistic (e.g. *F*, *t*, *r*) with confidence intervals, effect sizes, degrees of freedom and *P* value noted<br>*Give P values as exact values whenever suitable.*
☒	☐	For Bayesian analysis, information on the choice of priors and Markov chain Monte Carlo settings
☒	☐	For hierarchical and complex designs, identification of the appropriate level for tests and full reporting of outcomes
☐	☒	Estimates of effect sizes (e.g. Cohen's *d*, Pearson's *r*), indicating how they were calculated

Our web collection on statistics for biologists contains articles on many of the points above.

Software and code

Policy information about availability of computer code

Data collection	Zoom 2018A v6.2.9200.0, Harmony imaging v4.9
Data analysis	Statistical tests were performed in GraphPad Prism v9.1.2 for all bar graphs, line graphs, and violin plots. Pathway analysis was performed in Enrichr.<br><br>FACS analysis was performed in FlowJo v10.7.1<br><br>Code for putamen snRNAseq analysis is written in R using Seurat package, MAST, and hdWGCNA package (0.1.1.9005 (2022-06-17)) functions for sample integration, expression analysis, and weighted gene co-expression network analysis .<br><br>Whole blood RNAseq: Data management, preprocessing and differential expression analysis were performed in Google Terra workspaces using R 3.6 and Python 3.7.In this analysis, we have used the raw counts' output of Salmon v0.11.3 (PMCID:PMC5600148) for the abundant transcript estimation. The Deseq2 R package (version 1.26.0) was used to identify differentially expressed genes among PD cases versus controls.<br><br>TargetALS data was analyzed on in OmicSoft ArrayStudio RNA-Seq analysis pipeline (version 10.1.1.3).<br><br>The Qiagen Ingenuity pathway analysis tool and Enrichr computational systems biology interface were used for pathway enrichment analyses for differentially expressed gene sets. All data preprocessing and analysis steps are fully described in Methods and recorded in R code notebooks.

CRISPR screen analysis: MAGeCK v0.5.9 and MAGeCKFlute v1.10.0 were used to analyze CRISPR screen results.

ScRNAseq analysis: Samples were analyzed using Cell Ranger V4, Seurat 4.0.6 in R version 4.0.3, UCell package version 1.1.0, scCustomize v0.7.0, and PHeatmap package version 1.0.12

For manuscripts utilizing custom algorithms or software that are central to the research but not yet described in published literature, software must be made available to editors and reviewers. We strongly encourage code deposition in a community repository (e.g. GitHub). See the Nature Portfolio guidelines for submitting code & software for further information.

Data

Policy information about availability of data

All manuscripts must include a data availability statement. This statement should provide the following information, where applicable:

- Accession codes, unique identifiers, or web links for publicly available datasets
- A description of any restrictions on data availability
- For clinical datasets or third party data, please ensure that the statement adheres to our policy

All data associated with this study are present in the paper or Supplementary Materials. AMP-PD data is available through the Terra platform (https://amp-pd.org/tools) by request and requires approval for access through the AMP-PD data use agreement. TargetALS datasets available through https://www.targetals.org/resource/genomic-datasets/. GRCh38 human reference genome available at https://www.ncbi.nlm.nih.gov/assembly/GCF_000001405.40. Transcriptomic data is available as cited, or accessible at synapse.org Synapse ID syn41699699 for the iPSC tri-culture processed gene counts matrixes. Fastq files are also available upon request at syn40800403.

Human research participants

Policy information about studies involving human research participants and Sex and Gender in Research.

Reporting on sex and gender	We report results for males and females, taking sex into account by the structure of our analysis. Sex information was provided by AMP-PD.
Population characteristics	The design formula used for each differential expression analysis included age group as a covariate.
Recruitment	Patients were recruited following procedures reported for each of the AMP-PD cohorts. www.amp-pd.org
Ethics oversight	The Access and Compliance Team membership is made up of representatives from the National Institutes of Health (NIH) and includes representatives from the National Institute of Neurological Disorders and Stroke (NINDS) and the National Institute on Aging (NIA). NINDS representatives are responsible for all access and compliance activities related to the AMP PD Unified Cohorts and NIA representatives are responsible for all access and compliance activities related to the Global Parkinson's Genetics Program (GP2) Cohorts.

Note that full information on the approval of the study protocol must also be provided in the manuscript.

Field-specific reporting

Please select the one below that is the best fit for your research. If you are not sure, read the appropriate sections before making your selection.

☒ Life sciences ☐ Behavioural & social sciences ☐ Ecological, evolutionary & environmental sciences

For a reference copy of the document with all sections, see nature.com/documents/nr-reporting-summary-flat.pdf

Life sciences study design

All studies must disclose on these points even when the disclosure is negative.

Sample size	No power analysis or other statistical analysis was used to determine sample sizes, but our sample sizes are simliar to other publications (Guttikonda et al., 2021; Zou et al., 2020)<br><br>Guttikonda, S. R., Sikkema, L., Tchieu, J., Saurat, N., Walsh, R. M., Harschnitz, O., … Studer, L. (2021). Fully defined human pluripotent stem cell-derived microglia and tri-culture system model C3 production in Alzheimer's disease. Nature Neuroscience, 1–12. doi:10.1038/s41593-020-00796-z<br><br>Zou, Y., Li, H., Graham, E. T., Deik, A. A., Eaton, J. K., Wang, W., … Schreiber, S. L. (2020). Cytochrome P450 oxidoreductase contributes to phospholipid peroxidation in ferroptosis. Nature Chemical Biology, 16(3), 302–309. doi:10.1038/s41589-020-0472-6
Data exclusions	No data was excluded from the analysis.
Replication	All data presented in this study is representative of at least three replicates, except for FACS analysis of tri-culture which was shown for

Replication	representative purposes. Genome-wide CRISPR screen was performed in duplicate. All attempts at replication were successful.
Randomization	Wells were randomly chosen on each plate for each condition. Flasks for treatment in the CRISPR screen were randomly chosen for each condition.
Blinding	Blinding was not relevant to our study as all measurements were automated such that bias would not be introduced.

Reporting for specific materials, systems and methods

We require information from authors about some types of materials, experimental systems and methods used in many studies. Here, indicate whether each material, system or method listed is relevant to your study. If you are not sure if a list item applies to your research, read the appropriate section before selecting a response.

Materials & experimental systems

n/a	Involved in the study
☐	☒ Antibodies
☐	☒ Eukaryotic cell lines
☒	☐ Palaeontology and archaeology
☒	☐ Animals and other organisms
☒	☐ Clinical data
☒	☐ Dual use research of concern

Methods

n/a	Involved in the study
☒	☐ ChIP-seq
☐	☒ Flow cytometry
☒	☐ MRI-based neuroimaging

Antibodies

Antibodies used	mouse anti-ferritin (RnD Systems MAB93541), clone 962609<br>rabbit anti-IBA1 (Wako 019-19741), polyclonal<br>chicken anti-MAP2 (abcam ab92434), polyclonal<br>chicken anti-GFAP (Millipore AB5541), polyclonal,<br>rabbit anti-SEC24B (Cell Signaling Technology 12042S), Clone D7D6S<br>mouse anti-β-actin (Sigma A5441), clone AC-15<br>mouse anti-GLAST (Miltenyi 130-118-344), clone ACSA-1<br>mouse anti-βIII tubulin (Millipore MAB1637), clone TU-20<br>rabbit anti-LC3B (Sigma L7543), polyclonal<br>guinea pig anti-p62 (MBL PM066), polyclonal,<br>donkey anti-mouse alexa 488 (invitrogen A21202), polyclonal<br>donkey anti-rabbit alexa 555 (invitrogen A31572), polyclonal<br>goat anti-chicken alexa 647 (invitrogen A21449), polyclonal<br>donkey anti-mouse 680 (Li-Cor 926-68072), polyclonal<br>donkey anti-rabbit 800 (Li-Cor 926-32213), polyclonal
Validation	ferritin – immunocytochemistry, see manufacturer's references<br>IBA1 - immunocytochemistry, see manufacturer's references<br>MAP2 - immunocytochemistry, see manufacturer's references<br>GFAP - immunocytochemistry, see manufacturer's references<br>SEC24B - immunoblot, see manufacturer's references<br>β-actin - immunoblot, see manufacturer's references<br>GLAST – Flow Cytometry, see manufacturer's references<br>βIII tubulin – immunocytochemistry, see manufacturer's references<br>LC3B – immunocytochemistry, see manufacturer's references<br>P62 - immunoblot, see manufacturer's references<br>donkey anti-mouse alexa 488 – immunocytochemistry, see manufacturer's references<br>donkey anti-rabbit alexa 555 – immunocytochemistry, see manufacturer's references<br>goat anti-chicken alexa 647 – immunocytochemistry, see manufacturer's references<br>donkey anti-mouse 680 - immunoblot, see manufacturer's references<br>donkey anti-rabbit 800 - immunoblot, see manufacturer's references

Eukaryotic cell lines

Policy information about cell lines and Sex and Gender in Research

Cell line source(s)	hipsc-derived microglia, neurons, and astrocytes were purchased from FujiFilm. Human immortalized microglia cell line was purchased from abmgood. HAP1 cell lines were purchased from Horizon Discovery. Lenti-X 293T cell line was purchased from Takara bio.
Authentication	CRISPR-edited SEC24B KO HAP1 cell lines were validated by sanger sequencing, qRT-PCR, and western blot. HiPSC-derived microglia, neurons, and astrocytes were validated by FujiFilm, see https://www.fujifilmcdi.com/products/neural-cells. In addition, cells were validated by immunocytochemistry and scRNAseq. Human immortalized microglia cell line was validated

by immunocytochemistry. Lenti-X 293T cells were not validated beyond what was provided by takara bio (https://www.takarabio.com/products/gene-function/viral-transduction/lentivirus/packaging-systems-and-cells/lenti-x-293t-cells).

Mycoplasma contamination	All cell lines tested negative for mycoplasma.
Commonly misidentified lines (See ICLAC register)	No commonly misidentified cell lines were used in this study.

Flow Cytometry

Plots

Confirm that:

☒ The axis labels state the marker and fluorochrome used (e.g. CD4-FITC).

☒ The axis scales are clearly visible. Include numbers along axes only for bottom left plot of group (a 'group' is an analysis of identical markers).

☒ All plots are contour plots with outliers or pseudocolor plots.

☒ A numerical value for number of cells or percentage (with statistics) is provided.

Methodology

Sample preparation	Cells were washed 2x with PBS. Cells were then treated with 0.25% Trypsin + EDTA (Sigma T4049 for 6min in 37°C 5% CO2 incubator. 1 volume of PBS + 2% FBS (Gibco A38400-01) was added to quench. Cells were combined from two wells and put through 40um cell strainers (BD Falcon 352235). Strained cell suspension was placed in a 1.7ml eppendorf tube on ice. Cell suspensions were spun at 4°C for 5min at 1500RPM. Supernatant was removed and cells were resuspended. Collected cells were resuspended in flow cytometry staining buffer (BD 554656) containing anti-GLAST (Miltenyi 130-118-344) PE-conjugated antibody for 10 minutes at room temperature. Samples were washed once, resuspended, and analyzed on a BD LSRII instrument.<br>For lipid peroxidation, collected cells were resuspended in FACS buffer (PBS + 1.5% BSA (Sigma A1470) + 5mM EDTA (Nalgene AM9260G) + 5% FBS (Sigma F2442)). Cell suspensions were placed through a 40µm cell strainer on a 5mL round-bottom tube (BD falcon 352235). Cells were processed on Sony SH800S cell sorter under the FITC and PE filters.
Instrument	LSRII, sony SH800S cell sorter
Software	FlowJo v10.7.1
Cell population abundance	No sorting. Collected approximately 15,000 events.
Gating strategy	Singlet events were selected based on forward scatter height and area.

☒ Tick this box to confirm that a figure exemplifying the gating strategy is provided in the Supplementary Information.
